# Mechanistic insights on irradiation induced fabrication of nanoporous *guar gum*/chitosan pH responsive hydrogels: a combined DFT and spectroscopic study

**DOI:** 10.1039/d6ra06085d

**Published:** 2026-10-20

**Authors:** Dhana Sai Shree K., Jogender, Eric W. Cochran, Sunita Rattan

**Affiliations:** a Amity Institute of Applied Sciences, Amity University Noida U.P. 201313 India srattan@amity.edu; b Pradhanmantri College of Excellence SLP Government Post Graduate College Morar Gwalior M.P. 474006 India; c Department of Chemistry, Iowa State University Ames IA 50011 USA

## Abstract

Gamma (γ) irradiation induced modification of biopolymers has emerged as a sustainable alternative to fabricate stimulus responsive hydrogels. Even though the energy driven technique promises an efficient and scalable method that can minimize chemical usage, utilization of renewable biopolymers like *guar gum* not only creates a circular economy, but also delivers a sustainable alternative to synthetic polymers. Although *guar gum*, is widely studied due to its biocompatible, biodegradable nature and exceptional pH- and temperature-dependent gelling properties, limitations due to uncontrolled swelling and low mechanical strength make it crucial to modify its polymer chains to create a functional material. To address these challenges, the current work establishes a clear mechanistic understanding of γ irradiation of *guar gum* as a crosslinker in *guar gum*–chitosan (GC) matrixes, which was supported through DFT and spectroscopic studies. This was done by pre-irradiating *guar gum* with an energy source (γ rays), which created reactive oxygen sites that could introduce crosslinks through radical recombination, which affected the swelling and mechanical properties of the hydrogel. Spectroscopic and computational studies showed that crosslinking is concentrated at the C6 and C4 positions of the mannose chain in irradiated *guar gum* units, where glycerol is incorporated as a flexible crosslinking bridge (R-O-R) at higher concentrations of γ-G rather than solely acting as a plasticizer. DFT calculations showed a favorable binding energy (−9.516 eV) for water elimination at the C6 position of the mannose unit which supported the feasibility of the reaction. The varying concentrations of γ-G (100, 200, 300, and 400 ppm) resulted in non–monotonic variance, where surpassing the critical concentration of γ-G in GCγ-400 led to the formation of a flexible, homogeneous network that controlled pH-responsive behavior and enhanced mechanical properties, unlike the rigid, brittle network dominated by backbone-backbone (R–R) crosslinks formed in GCγ-300. While the pore radii remained comparable across the formulations (∼1.5 to 2.2 nm), GCγ-400 showed the greatest increase in surface area (15.9 to 33.7 m^2^ g^−1^) and pore volume (0.024 to 0.049 cm^3^ g^−1^) relative to pristine GC, suggesting its potential for stimulus responsive based applications like sensors, actuators, drug delivery systems *etc.* Therefore, the study demonstrates that pre-irradiated polysaccharide can function as a macromolecular radical crosslinking initiator where a varying concentration of γ-G modulated radical recombination induced crosslinking within the respective polymer matrix.

## Abstract 2

Fabrication of stimulus responsive nanoporous hydrogels by modifying *guar gum* through γ rays, *via* concentration dependent radical crosslinking recombination (varying γ-G) in *guar gum*–chitosan, due to flexible crosslinks formed at highest concentration.

## Graphical abstract

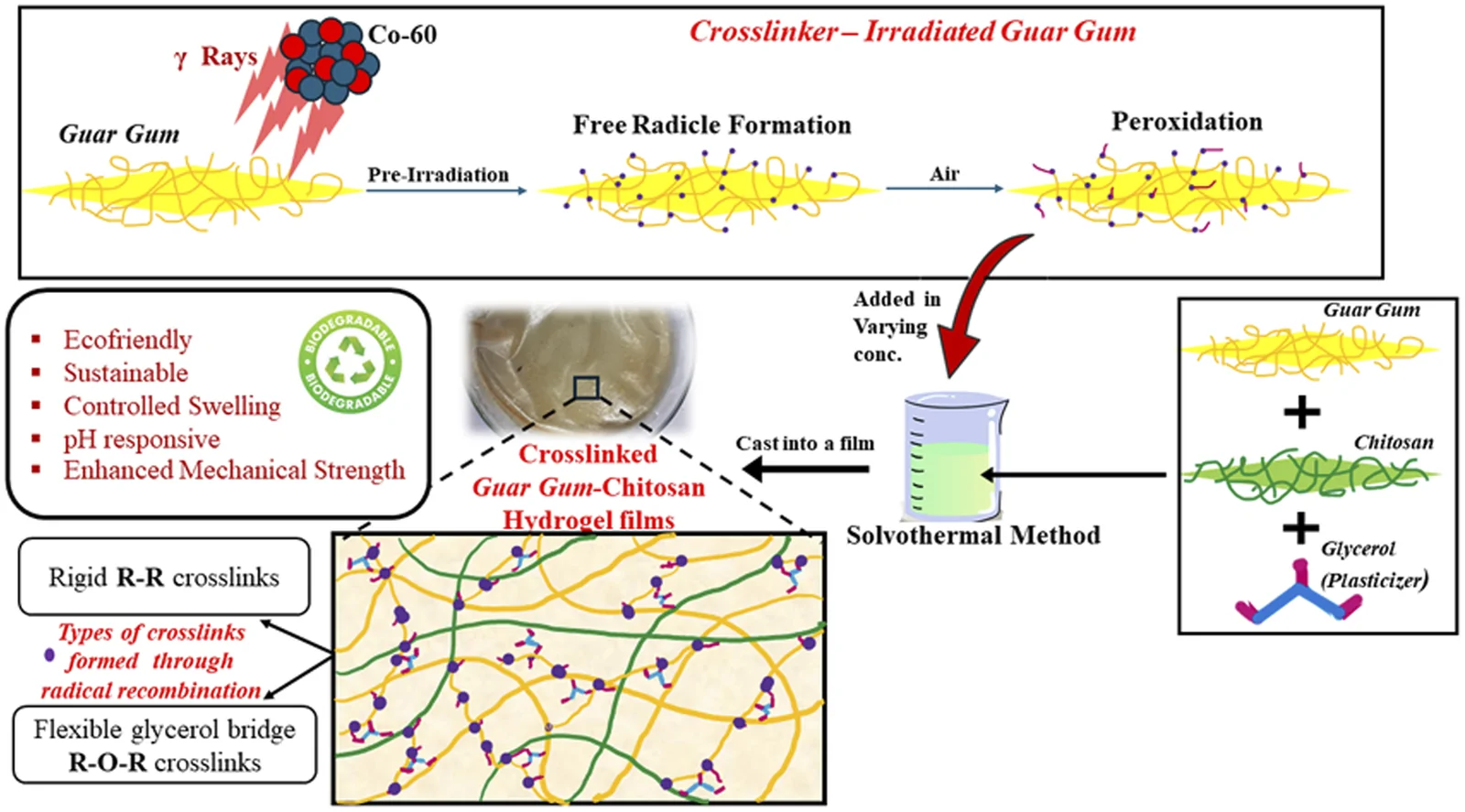

## 1. Introduction

Biopolymers are a green, sustainable alternative to synthetic polymers, as they are highly biodegradable, biocompatible, and abundantly available. Polysaccharides like *guar gum* are a class of such biopolymers that show alteration in their viscoelastic properties in response to pH, temperature, *etc.* Although *guar gum* can act as a potential emulsifier, its use in its pristine form is limited by excessive swelling capacity, low mechanical strength, and rapid biodegradability. To overcome these limitations, it is crucial to modify the *guar gum* chains to make them applicable in various fields. The abundant OH groups in *guar gum* both provide convenient sites for chemical modification and tend to interact physically with other polymers and functional moieties.^1^ These modifications include grafting, crosslinking, functionalization of other inorganic or organic groups *etc.* through physical or chemical reactions that allow biopolymers to be used in various applications.^2,3^ Crosslinking is a modification that introduces a crosslinker between polymeric chains, resulting in polymer branching and altering the polymer's physicochemical properties.^4^ Studies have revealed that the crosslinking density determines mechanical properties, stimulus-responsive behavior, and thermal properties of the polymers as shown by Y. Guan *et al.* and R. Zhu *et al.*^5,6^ The crosslinking density depends on the concentration of the crosslinker, which regulates branching between polymer chains to modify them for their specific application, as stated by H. V. Chavda *et al.*^7^ As physical methods show weak crosslink formation, chemical crosslinking techniques are widely being explored. Some readily commercialized synthetic crosslinkers include tetraethoxysilane (TEOS), tetraethyl orthosilicate, and sodium tripolyphosphate (Na-TPP); these have disadvantages such as environmental impact, and complex purification processes, more benign methodologies for stimulus responsive based applications.^8,9^

As an alternative, efforts have been made to modify biopolymers using irradiation techniques, in which biopolymers are subjected to energy sources such as microwaves, UV, gamma (γ) radiation, or ion beams to create reactive sites that facilitate potential crosslinking of polymer chains. Y. S. Hamed *et al.* reported that the alteration of the polysaccharide using γ irradiation induces chain scission and crosslinking between the polymer chains, affecting their physicochemical properties.^10^ R. Sabry *et al.* synthesized a pectin-based biofilm using the electron beam irradiation technique, where the concentration of pectin, along with irradiation, affects the mechanical properties of the film.^11^ Polysaccharides can be subjected to γ rays in two different ways, *i.e.*, mutual and pre-irradiation, where C˙ radicals are generated at specific sites depending on the dosage that react with atmospheric O_2_ to form C–OO˙ reactive oxygen sites that aid in crosslinking, according to the study done by D. Ballentine *et al.*^12,13^ To initiate radical polymerization, various factors like radiation dose, temperature, solvent *etc.* assist in radical induced recombination in the polymer matrix which modifies the polymer chains either through crosslinking or grafting.^14–16^ For instance, J. Biswal *et al.* had mutually irradiated *guar gum* and acrylamide where it was observed that increase in radiation dose increased the grafting ratio due to greater radical generation.^17^ On the other hand, T. T. Hanh *et al.* grafted polyacrylonitrile on pre-irradiated biopolymer where the elevated reaction temperature activated the trapped radicals that initiated radical polymerization.^18^ Recent studies have also shown that γ ray induced modification of biopolymers can tailor the network structure that influence the stimulus responsive behavior of the biopolymers.^19–21^ The present work, therefore, focuses on modifying biopolymers, such as *guar gum*, *via* γ-ray pre-irradiation to create crosslinks through concentration dependent radical recombination in biopolymers that affect both swelling and mechanical properties. Unlike prior approaches that rely on mutual irradiation with an external crosslinker or grafting of a separate functional moiety, the present work uses γ-irradiated *guar gum* (γ-G) itself as the active crosslinking species within the matrix where glycerol is selectively incorporated as flexible bridging unit at higher concentration of γ-G.

*Guar gum*, a type of polysaccharide, is derived from the endosperm seeds of *Cyamopsis tetragonolobus*^22^, consisting of galactose (36.6%) side chain and mannose (63.1%) long chain.^23^ It has a major advantage in pre-irradiation-induced modification due to the sterically available C4 and C6 positions of the mannose unit, which facilitate the easy abstraction of hydrogen to form C˙ radicals that can undergo peroxidation.^24,25^ Apart from that, the galactose monomer orients the polysaccharide chain, thereby restraining chain-to-chain interactions, altering its flexibility, and increasing toughness. However, due to high-capacity branching, interchain hydrogen bonding results in a positive response in hydration properties.^26^ The most important aspect that allows *guar gum* to be considered for pre-irradiation technique is its tendency to undergo controlled radical scission in its galactomannan chain that yields active macro-radical sites upon which peroxidation can act as potential crosslinking initiators in the hydrogel matrix.^27–29^ Other reasons that make *guar gum* a potential candidate to be considered in this category are its renewable nature, anti-inflammatory, intensive swelling, altering viscosity and hydration capacity depending on the temperature, pH, *etc.*, apart from high stability in a wide range of pH.^1,30,31^ This makes it commonly used as an emulsifier and thickening agent in the pharmaceutical and food industry. R. Yousry *et al.* chemically modified *guar gum* and incorporated polyacrylic acid into its chains using γ irradiation for the fabrication of a hydrogel as a remediation for dye removal. To control the high hydrophilicity of *guar gum*, a hydrophobic moiety (polyacrylic acid) was incorporated *via* γ-irradiation-induced crosslinking, making it applicable as a hydrogel.^32^ Studies have also showed that depending on the modification and incorporation of different moieties in *guar gum*, stimulus responsive materials could be fabricated. For *e.g.*, Zhang *et al.* developed a pH responsive hydrogel by crosslinking chitosan and *guar gum* with PVA, which not only showed accelerated release of the antifungal agent but also improved its photostability and antifungal efficacy.^33^ Apart from that, Santhamoorthy and Kim reviewed recent advances in biopolymer hydrogel based scaffolds fabricated through crosslinking and grafting for drug delivery and tissue engineering which highlighted the challenge of balancing mechanical strength and flexibility with tunable stimuli responsive behavior.^34,35^

Imparting hydrophobicity to the *guar gum* matrix can also be achieved by using another class of polysaccharides, such as chitosan, which, depending on the type of interactions, can impart hydrophobicity, making it a completely green, sustainable material.^36^ Chitosan, which is a derivative of chitin, consists of d–d-glucosamine and *N*-acetyl-d-glucosamine units.^37,38^ Although protonation of the –NH_2_ group in the glucosamine unit of chitosan makes it soluble in water, studies done by S. Rahman *et al.* suggested that *guar gum* and chitosan, when crosslinked, imparted hydrophobicity with alteration in swelling and mechanical properties of the hydrogel.^38^ The NH_2_ group also imparts pH-responsive behavior in the material, which makes *guar gum* and chitosan preferred natural materials to synthesize stimulus-responsive hydrogels.^30,39^ Apart from controlled swelling, mechanical properties are another parameter that needs to be enhanced in *guar gum* for further applications. In order to make the biopolymer-based films flexible and mechanically stable, plasticizers such as glycerol are used due to their functional OH groups that incorporate in between the chains, so that it can reduce the interchain interactions that help in the formation of crosslinks.^40^ Other than plasticizer, crosslinking also plays a role in enhancing the mechanical properties of the biopolymers.^41^ Toni D. Novianto *et al.* stated that when optimal crosslinks are formed due to gamma irradiation, the mechanical properties also enhance, depending on the dosage.^42^

Hence, in this work, a unique strategy has been used to structurally modify *guar gum via* γ-irradiation using a pre-irradiation technique to crosslink the *guar gum*–chitosan (GC) matrix and fabricate a nanoporous, pH-responsive hydrogel with controlled swelling and enhanced mechanical strength. In the current work, the addition of γ-modified *guar gum* (γ-G) serves as a macromolecular radical crosslinking initiator where varying its concentration modulated radical recombination in the polymer matrix. When γ-G was added at varying concentrations to the *guar gum*-chitosan moiety with glycerol, as a plasticizer, glycerol not only acted as a passive plasticizer but it also incorporated itself as a flexible crosslinking bridge at sufficiently high γ-G concentration (400 ppm), where physicochemical modifications were observed, suggesting that the concentration driven radical recombination *via* γ pre-irradiation technique can be used to induce potential crosslinks. The *in silico* analysis identified potential modified sites in γ-irradiated *guar gum* (γ-G) that could provide reactive sites to interact with GC bases and act as crosslinker. The results demonstrate that pH-responsive behavior, mechanical properties, hydrophilicity, and the formation of a nanoporous matrix can be precisely tuned by controlling the concentration of irradiated *guar gum* to determine radical recombination dynamics.

## 2. Experimental

### 2.1. Materials

All chemicals and reagents were of analytical grade and were used without further purification. *Guar gum* powder of endosperm, chitosan (extra pure), and glycerol were procured from Merck, India. Glacial acetic acid was procured from Emplura Solutions, India.

### 2.2. γ irradiation-induced modification and fabrication of hydrogel film

*Guar gum* and chitosan composite with varying concentrations of the crosslinker were synthesized according to the procedure followed by M. U. A. Khan.^43^ First, *guar gum* in the dry state was γ irradiated Indian National Gamma Array (IUAC, New Delhi) at different dozes (2–10 kGy) having the dose rate of 1.35 Gy min^−1^ to fabricate γ-G. After which 1% w/v chitosan was dissolved in 2% acetic acid solution and stirred for 2 h. This was added dropwise to the 2% w/v *guar gum* solution, which was stirred for 1 hour. Afterward, 1% w/v of glycerol was added, maintaining the above conditions. A separate solution of γ-G with varying concentration (100–400 ppm) prepared, was added immediately dropwise to the above mixture, which was stirred at 400 rpm for 3 h at 60–80 °C (ref. 18) (Fig. 1).

**Fig. 1:**
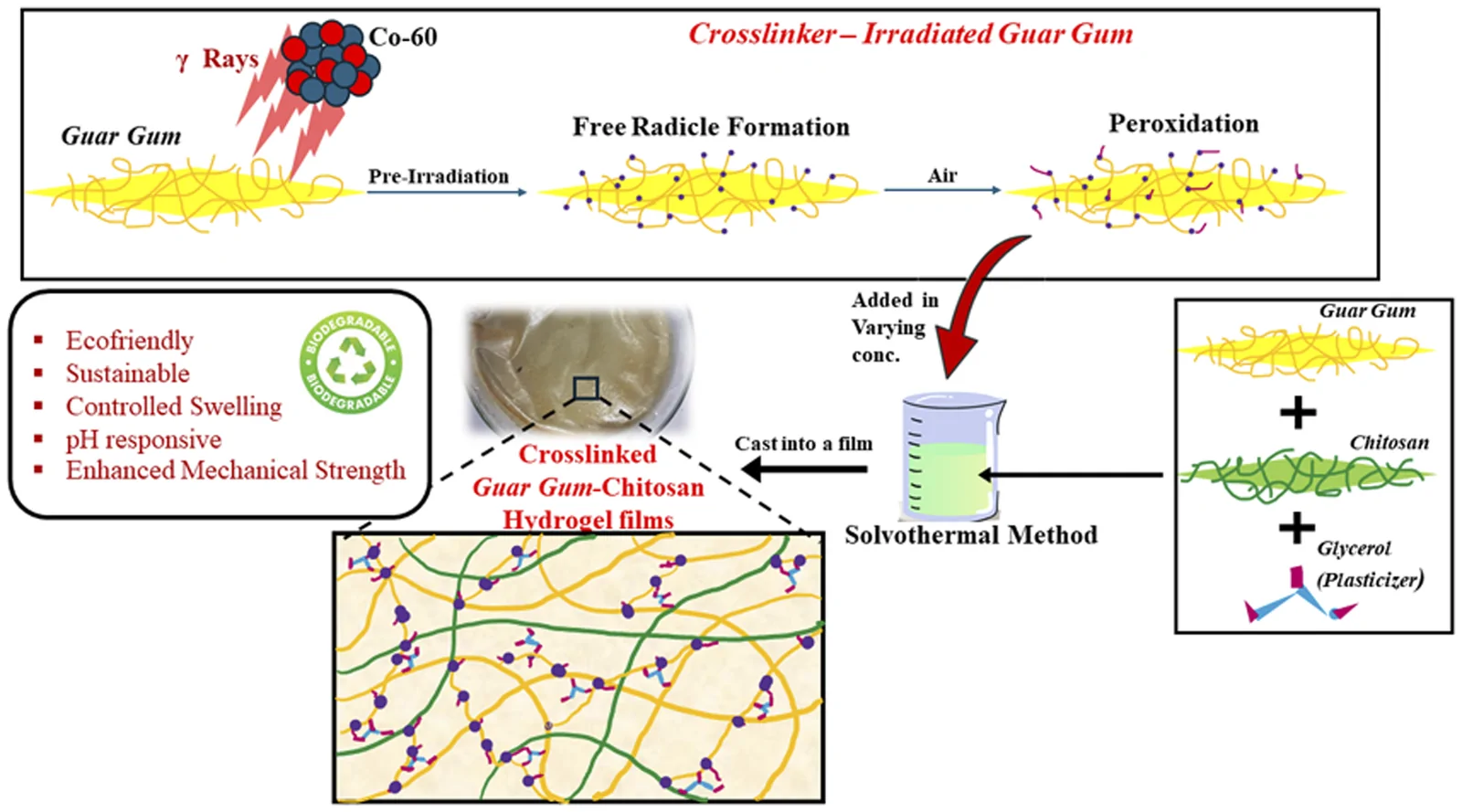
Schematic representation of effect to fabricate a crosslinked *guar gum*–chitosan stimulus responsive hydrogel.

### 2.3. *In silico* analysis of the interaction of γ-irradiated crosslinker with polymer chains

DFT calculations were performed using DMol^3^ (ref. 44 and 45) with a double numerical basis set including polarization functions (DNP) and DFT semicore pseudopotentials (DSPP) for core electrons.^46^ Exchange-correlation effects were treated using the GGA-PBE functional^47,48^ with long-range dispersion accounted for *via* Grimme's DFT-D correction.^49^ Calculations were spin-unrestricted, using a Fermi smearing of 0.02 Ha, and results were extrapolated to 0 K.^50^ Geometry optimizations employed delocalized internal coordinates with convergence thresholds of 2.0 × 10^−5^ Ha (energy), 4 × 10^−3^ Ha/Å (force), and 5 × 10^−3^ Å (displacement). Hirshfeld population analysis was used for partial charges^51^ and Mayer's formalism for bond orders.^52^

### 2.4. Characterization

#### 2.4.1. ATR-FTIR for molecular interaction

The molecular interactions between the polymer chains can be studied using attenuated total reflectance–Fourier transform infrared spectroscopy (ATR-FTIR). A Shimadzu spectrophotometer was used to obtain spectra over 500–4000 cm^−1^, which helped identify structural differences between pure *guar gum* and irradiated *guar gum* and proposed the mechanistic pathway (Scheme 1).

**Scheme 1:**
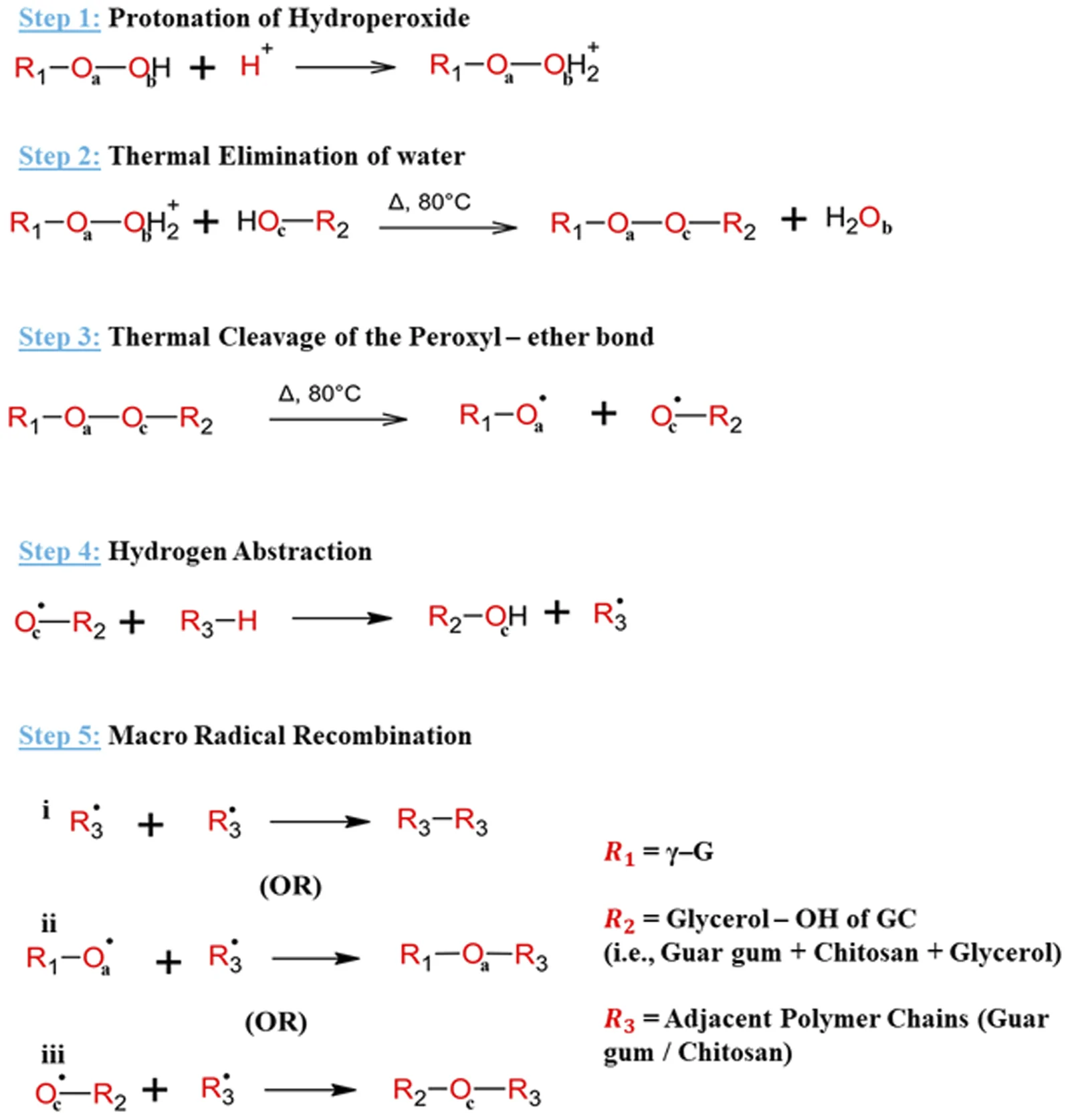
Mechanistic insights on the radical recombination induced crosslinking in the hydrogel formulations that determine the physiochemical properties of the polymer matrix which are affected by the type of crosslinks that dominate in the hydrogel films.

#### 2.4.2. ^13^C NMR analysis for structural information on possible crosslinking sites

^13^C solid–state NMR was recorded for GC and GC-γ-G on a JEOL ECX-400II at 10 000 Hz with a 4 mm rotor. The number of scans performed on the respective samples was approximately 1000, which provided the chemical shift (*δ*) values that were reported in ppm.

#### 2.4.3. Colorimetric analysis for determination of glycerol conc

In order to determine the concentration of glycerol involved with γ-G, colorimetric analysis was carried out by the Malaprade and Hantzsch reactions where the amount of free glycerol in each formulation was determined. This methodology allowed NaIO_4_ in Malaprade reaction to first oxidize glycerol to form formaldehyde which in turn reacted with the Nash reagent in Hantzsch reaction to from 3,5-diacetyl-1,4-dihydrolutidine (or DDL) which was a yellow-colored dye that gives the *λ*_max_ at 410–412 nm.^53–55^ At first 50 mg of the hydrogel films were washed with ethanol water mixture at the ratio of 1 : 1 and then sonicated at 70 °C for 2 hours in 5 mL D.I. water. After obtaining the standard graph through the above reactions, 1 mL of the sample extract was taken and mixed with Malaprade reagent in the dark for 15 min at room temperature (R.T). 2 mL of Nash reagent was then added to the above mixture and then stirred in the water bath at 50 °C for 15 min to develop a bright yellow color after which the solutions were cooled to R.T. and the absorbance was measured where using the linear regression equation the concentration of the free glycerol was determined.^56^

#### 2.4.4. Swelling analysis (in water and pH) and their kinetics

Swelling studies were performed on vacuum-dried hydrogel films (2 × 2 cm^2^) in different solvent systems, *i.e.*, DI water and phosphate buffer solutions (pH 4, 7.5, and 9). The weight of the swollen hydrogel was then recorded at regular intervals and further analyzed to determine the swelling percentage and its kinetics. Varying pH (4, 7.5, 9) using phosphate buffer was selected in order to observe the pH responsiveness of the material.^57^ The swelling ratio was determined using the formula:1
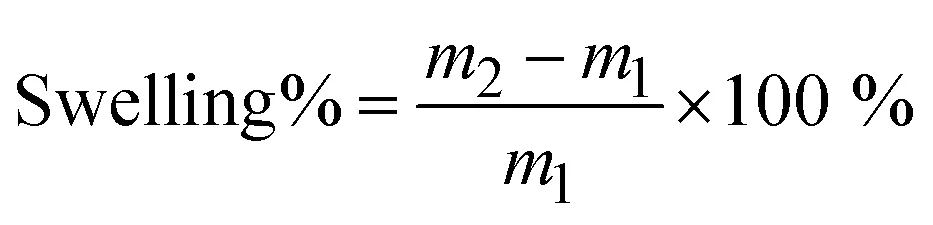
where *m*_1_ is the weight of the dry hydrogel, and *m*_2_ is the weight of the wet hydrogel composite.

The swelling data were fitted to Peppa's model to determine the diffusion mechanism and reaction order. The diffusion parameters along with the type of diffusion can be obtained by Ritger-Peppa's equation, *i.e.*,

2 *F* = *kt*^*n*^

3 ln *F* = ln *k* + *n* ln *t*

where *F* is the fractional swelling, which is the weight of the swelling at time *t* divided by the swelling weight at equilibrium time; *k* is the swelling rate constant, and the value of *n* helps determine the solvent transport pathway in the hydrogel film. The swelling data for hydrogels in DI water were used to plot ln *F vs.* ln *t*, and the values of *k* and *n* were determined from the graph to yield the diffusion parameters. The transport mechanism for *n* ≤ 0.5 is termed as Fickian and, whereas *n* ≥ 0.5 is termed as Non-Fickian^58^ and the values well below the threshold are classified as quasi-Fickian which states that the diffusion process is faster than polymer chain relaxations.^58^

The first and second-order swelling kinetics was calculated by the following equations, where the highest correlation value (*R*^2^) determined the swelling behavior of the hydrogel. The first order equation, which states that the rate of water adsorbed depends on the concentration gradient, is as follows:^59^4
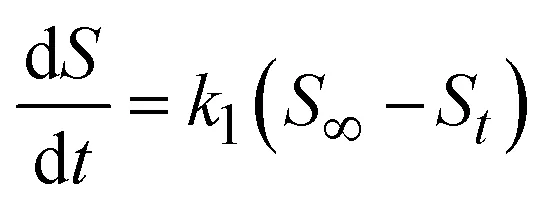
where *S*_∞_ is the degree of swelling at equilibrium, *S*_*t*_ and degree of swelling at time ‘*t*’, and *k*_1_ is the first-order rate constant. After integrating and applying the conditions of *S* = 0 at *t* = 0 and *S* = *S* at *t* = *t*, the equation becomes,

5 ln *S* = *k*_1_*t*

6
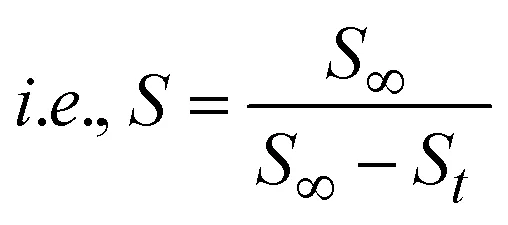


The equation for the second-order kinetic equation, which depends on the polymer unit, is as follows:^59,60^7
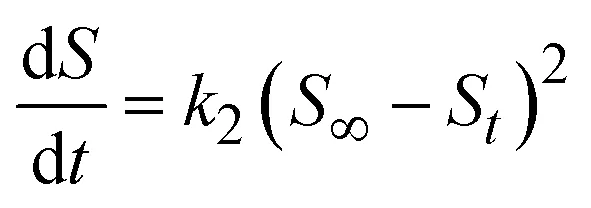
where *k*_2_ is the second-order rate constant. On further integrating the equation following the same above conditions, the corresponding equation can further be written as:8
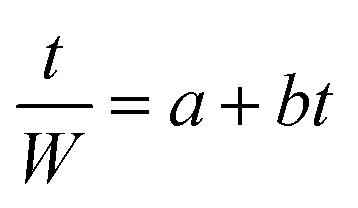
where *a* is the inverse of the initial swelling and *b* is the reciprocal of the swelling rate at equilibrium.9
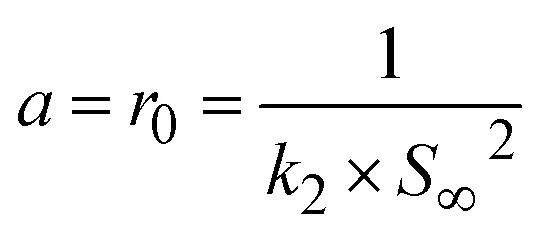
10
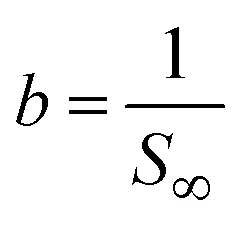


#### 2.4.5. FESEM for morphological studies

FESEM (Zeiss GeminiSEM 500- Thermal field emission type) was used to analyze the morphology of GC and GCγ hydrogel films. The sample was coated with gold, then placed on the disc using carbon tape.

#### 2.4.6. Contact angle for wetting analysis

A contact angle analyzer (SEO Instruments) was used to determine how varying concentrations of the crosslinker affected the wetting properties of the hydrogel films. 10 µL of D.I. water was dropped on the vacuum-dried film to measure the contact angle of each sample at the repetition of 50 to obtain M ± SD values. As the crosslinked hydrogel films showed hydrophobicity despite the hydrophilic nature of the crosslinker, the Cassie–Baxter effect was studied for GCγ-400 formulation, which states that a liquid droplet rests on a textured surface, with air trapped between the liquid and the solid surface. The Cassie–Baxter equation eqn (11) was solved to determine the percentages of solid and air surfaces that rest under the water droplet during the wetting analysis.

11 cos *θ*_*m*_ = *f*_1_*θ*_*i*_ − *f*_2_

where *f*_1_ + *f*_2_ = 1, *θ*_*m*_ is the experimentally measured contact angle on solid air gaps, *θ*_*i*_ is the equilibrium constant angle on the flat surface of the same solid, *f*_1_ is the total area of the solid under the drop per unit projected area, and *f*_2_ is the total area of air under the drop per unit projected area.

#### 2.4.7. Brunauer–Emmett–Teller (BET) method for understanding the porosity of the hydrogel film

The surface area, pore volume, and pore size of the GC and GCγ were analyzed using the Brunauer–Emmett–Teller (BET) Surface Area Analyzer (Quantachrome instrument version 5.2), which used the Barrett–Joyner–Halenda (BJH) method for pore size and volume analysis. The samples were prepared by degassing overnight at 50 °C under vacuum, and the pore size distribution was determined using the BJH method.

#### 2.4.8. UTM analysis for mechanical properties

Mechanical properties such as Young's modulus, tensile strength, elongation at break, and toughness were obtained by using a universal testing machine (Zwick Roell, Germany Static UTM Z010) according to ASTM D-882 standard test (ASTM, 1992) at room temperature in a dry state. Rectangle-shaped samples measuring 6 cm × 1 cm were used to evaluate these properties.

## 3. Results and discussion

### 3.1. Schematic

The plausible chemical mechanism for the interaction of the crosslinker, *i.e.*, the γ-G unit, with the GC moiety was proposed in Scheme 2 along with the stepwise mechanism for radical recombination in Scheme 1 based on thorough literature, experimental, and *in silico* analyses. As stated earlier, D. Ballentine *et al.* showed that irradiating polymers in air led to the formation of reactive peroxide species.^13^ This indicated that irradiating *guar gum* units with γ rays could lead to the formation of a wide number of C˙ free radicals, but the most stable C–OO˙ reactive species were formed at C6 and C4 positions on the mannose unit due to easy hydrogen abstraction because of their steric availability, supporting the proposed the structure of γ-G.^24,25^ Per the mechanism proposed in crosslinked *guar gum* by R. S. Banegas *et al.*, the hydroxyl group of the C6 position in the galactose side chain also showed hydrogen bonding with glycerol because of the primary alcohol^61^ apart from the most reactive C6 position of the mannose unit as proposed by the mechanistic scheme of T. A. Mir *et al.*^62^ Regarding chitosan, although the NH_2_ group was the most reactive site, glycerol also formed hydrogen bonds with the CH_2_OH group, as reported by E. Rohaeti *et al.*^63^ This suggests that a possible crosslink between γ-G and the GC unit forms *via* the C6 and C4 positions. The chemical structure, when analyzed experimentally by IR and NMR, predicted that although C˙ radicals could form at all possible sites in γ-G, the most stable sites were C6 and C4. These carbons led to the formation of peroxides that reacted with GC *via* the glycerol molecule, as discussed in detail in later sections. As two C6 positions with primary alcohols, *i.e.*, one in the galactose unit and the other in the mannose unit of the *guar gum* chain, are present, energy optimization of γ-G suggested that the possible C6 position that formed a stable reactive peroxide was in the mannose unit, which further induced crosslinks with the GC unit.

**Scheme 2:**
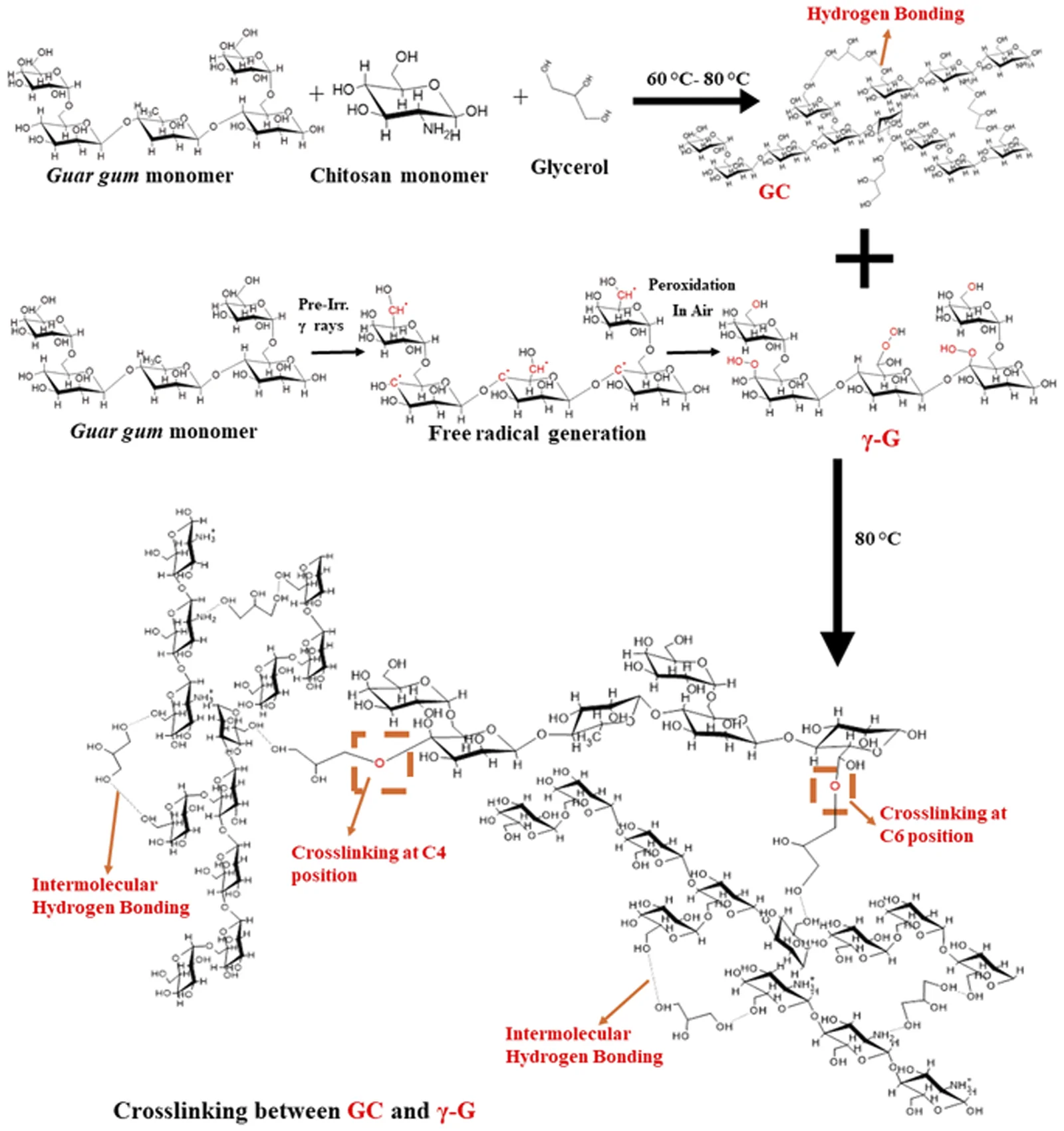
Proposed chemical reaction between crosslinking of irradiated material with *guar gum* chitosan moiety.

To further understand the mechanism and the behavior of the different formulations especially GCγ-400, Scheme 1 has been proposed for the stepwise formation of the crosslinks. At first, the biopolymer based hydroperoxide (γ-G) was protonated due to the presence of glacial acetic acid that was consistent with the study done by Seubold *et al.* in which acid-catalyzed activation of hydroperoxide functionalities was described for cumene hydroperoxide where protonation of –OOH groups is the initiating step in Brønsted acid conditions.^64^ This protonated hydroperoxide then undergoes acid-catalyzed water elimination which generated electrophilic oxygen center that was susceptible to nucleophilic attack by the OH groups forming the proxy ether linkage (R_1_–O–O–R_2_). This step was supported by the study done by Bityukov *et al.* where acid-catalyzed reactions of alcohol with hydroperoxide proceeding *via* water elimination to form new alkyl peroxide bonds was reported^29^ and also with the established role of Brønsted acid in catalyzing new bond formation as reported by Yaremenko *et al.* and Klussmann respectively.^65,66^ Additionally, DFT calculations and solid state ^13^C NMR also further supported this pathway (Section 3.2 and 3.3 respectively) showing a favorable binding energy of −9.516 eV through water elimination (OH from –OOH and H from –OH) at C6 position of the mannose unit of *guar gum* along with peaks at C4 and C6 positions of the crosslinked material at these two proposed positions. The newly formed peroxy–ether bond then undergoes thermal O–O cleavage that generated macroradicals on the biopolymer and glycerol fragments which is widely documented in radical peroxidation chemistry^28^ along with the acid catalyzed conditions which further facilitated radical generation from peroxide intermediates even near ambient temperature as stated by Klussmann.^66^ These generated radicals then abstract hydrogen atoms from adjacent hydroxyl or methine or methylene bearing positions in *guar gum*, chitosan or glycerol which is a well-studied pathway established by Bityukov *et al.* which generated secondary macroradicals distributed across the polymer matrix. These subsequent macroradicals then recombine forming new linkages like C–C (R–R) or C–O–C (R–O–R) at a sufficiently high concentration of γ-G between biopolymer backbone or glycerol which correlated with the radical coupling pathways described by Bityukov *et al.*^28^ These recombination steps constitute the plausible crosslinks formed which are responsible for network formation depending on the dominant crosslinking either backbone (R–R) or glycerol bridged (R–O–R) crosslinking with γ-G effected the properties of the hydrogel formulations.

The quantitative concentration-dependent retention of glycerol through the colorimetric analysis, together with complementary structural and mechanical evidence (mentioned in detail in the below sections) supports the involvement of glycerol with γ-G (Fig. 2). In pristine GC the high baseline of glycerol content (1.307 mg) was due to the extensive hydrogen bonding with the free biopolymer reactive sites of *guar gum* and chitosan which allowed glycerol to freely interpenetrate and bind with the polymer chains. On adding γ-G in varying concentration, the amount of bound glycerol varied in each formulation due to the reorientation of the polymer chains due to the formation of crosslinks. In GCγ-100 the bound glycerol slightly drops to 1.231 mg in comparison to GC which was due to the primary crosslink nodes formed due to radical recombination that react with the biopolymer active sites which reduced the availability for glycerol to be physically hydrogen bound with them. On the other hand, although γ-G concentration was increased, GCγ-200 showed the least amount of glycerol bound (1.045 mg) which was because the addition of γ-G not only altered the surface polarity of the polymer chains as reflected in contact angle measurements (highest hydrophilicity) but also increased crosslinking activity with increase in concentration of γ-G competed with the glycerol molecules that resulted in excess glycerol out of the bulk matrix. But as the concentration increased in GCγ-300 and GCγ-400, the addition of γ-G in GCγ-300 lead to severe chain entanglement and localized compaction which can be observed from the drastic drop in surface area and pore volume in BET analysis which restricted the network porosity and also explains its increased Young's Modulus and sharp drop in its elongation at break and toughness which is a characteristic of a rigid entangled network that is dominated by backbone–backbone (R–R) crosslinks (Scheme 1 (i)). This can be attributed to the physical entrapment of glycerol which corresponds to its 1.211 mg value that is higher than GCγ-200 but lower than GCγ-100. But in GCγ-400, as it crossed the critical concentration of γ-G, the involvement of free OH groups and adjacent hydrogens in radical recombination becomes much easier. This is because the increase in the –OOH groups reorient the matrix in such a way that the OH and H groups of *guar gum*, chitosan or glycerol are close to the excessive –OOH groups which led to the involvement of glycerol (1.409 mg bound) in crosslink formation (Scheme 1 (ii and iii)). This is directly reflected on GCγ-400 properties where the BET analysis showed the highest surface area and pore volume and a drop in Young's Modulus along with increase in elongation at break and toughness compared to other formulations. This combination of higher porosity, lower stiffness and greater flexibility was due to the radical recombination that incorporated glycerol as the flexible crosslinking bridge (Scheme 1 (ii or iii)) compared to the R–R crosslinking junction formed in GCγ-300. Apart from BET and mechanical properties, GCγ-400 also showed increase in hydrophobicity in contact angle along with high amount of glycerol bound to the polymer matrix which states that the crosslinks formed consumed more OH groups in radical recombination which led to less polar surface groups and more incorporation of glycerol in its matrix.

**Fig. 2:**
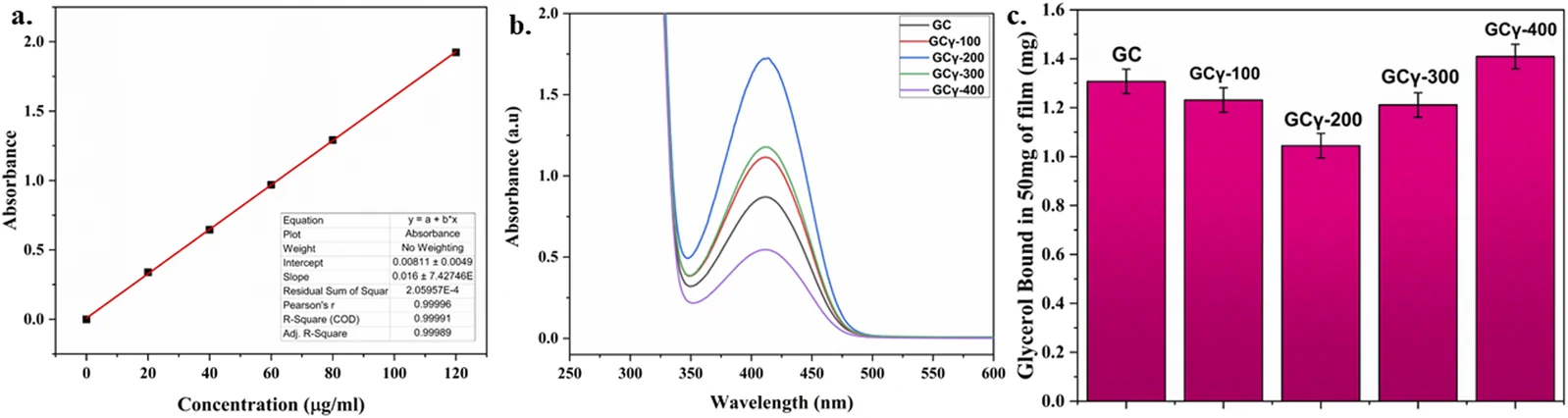
Colorimetric analysis to determine the amount of glycerol bound to 50 mg of hydrogel films. (a) Calibration curve of absorbance *vs.* concentration of glycerol, (b) absorbance of free glycerol in the respective hydrogel formulations, (c) amount of glycerol bound to 50 mg of hydrogel films.

### 3.2. *In silico* analysis using DFT

#### 3.2.1. Energy optimization of the geometries

Density Functional Theory (DFT) calculations were conducted using the DMol^3^ module,^44,45^ a component of the BIOVIA Materials Studio suite. Electronic structure computations employed a double numerical basis set augmented with polarization functions (DNP+), ensuring a balanced treatment of valence orbitals and polarization effects. DFT semicore pseudopotentials (DSPP) were applied to account for relativistic effects and to model the core electrons efficiently.^47^ Exchange–correlation interactions were described within the Generalized Gradient Approximation (GGA) using the Perdew–Burke–Ernzerhof (PBE) functional.^48^ To accurately capture non-covalent interactions, particularly long-range dispersion forces, Grimme's empirical dispersion correction (DFT-D) was incorporated into the total energy formalism.^49,50^ The binding energy (*E*_BE_) was evaluated using the standard supermolecular approach as follows:

12 *E*_BE_ = *E*_Complex_ − *E*_1_ − *E*_2_

where *E*_Complex_ denotes the total energy of the optimized complex comprising GC and γ-G and *E*_1_ and *E*_2_ represent the total energies of the individually optimized monomeric units, respectively.

All geometries were fully optimized to their respective local minima on the potential energy surface. The optimized structures of GC and γ-G are depicted in Fig. 3. Hirshfeld charge distributions and Mayer bond order analyzes for both units are provided in the SI to elucidate the electronic structure and bonding characteristics.

**Fig. 3:**
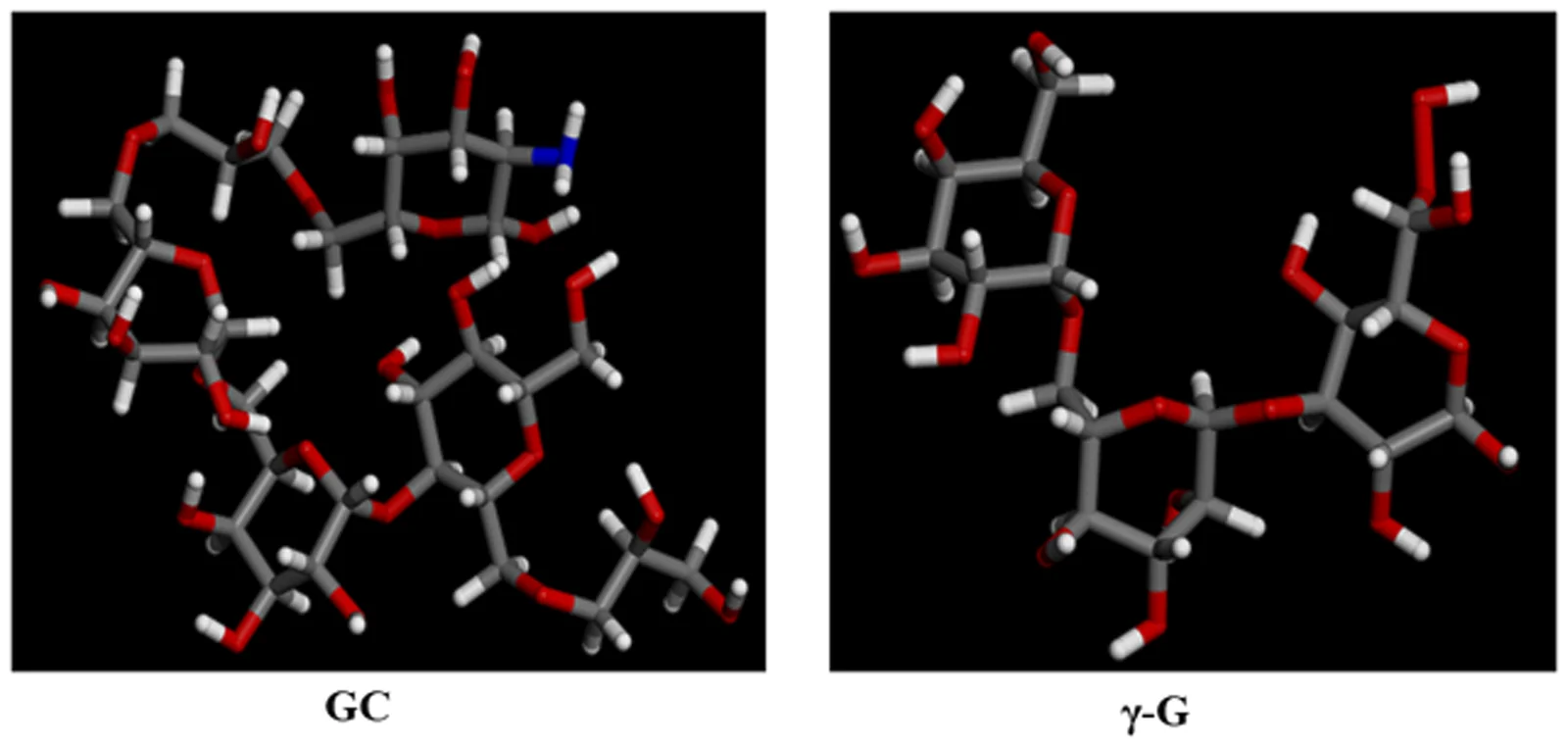
Optimized structure of GC and γ-G.

Frontier molecular orbital (FMO) analysis was also performed to assess the electronic properties of the ligands in both units. Table 1 summarizes the computed HOMO–LUMO energy gaps, indicating that GC possesses the largest energy separation. The spatial distribution of the frontier orbitals was visualized, with blue isosurfaces indicating regions of electron density accumulation and yellow isosurfaces indicating regions of electron density depletion.

**Table 1:** HOMO–LUMO gap for GC and γ-G

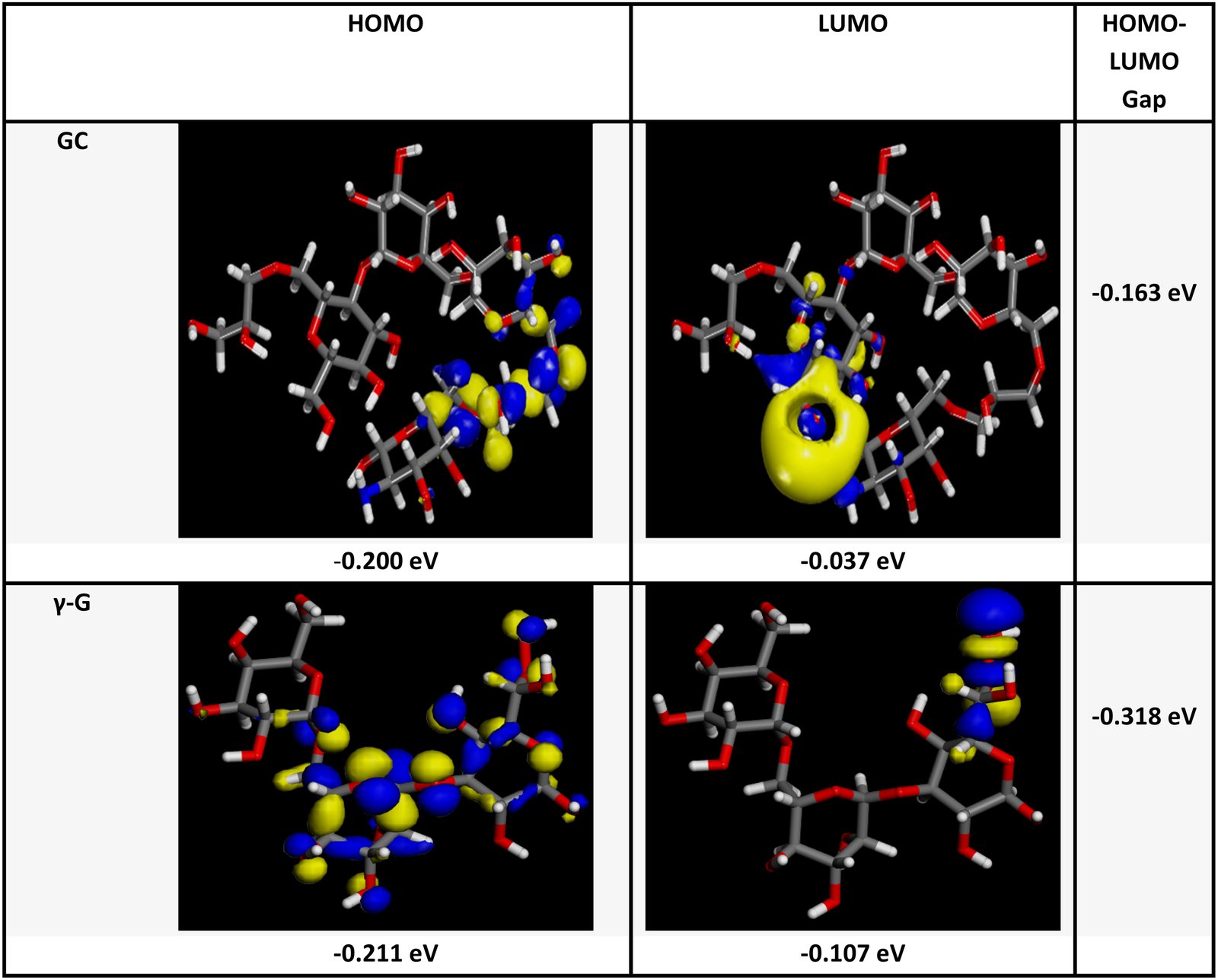

#### 3.2.2. Interaction of GC with γ-G

Further, we investigated the interaction between ligand GC and γ-G by performing adsorption locator calculations to identify the most favorable adsorption configurations. Among the computed adsorption geometries, the first three modes were analyzed in detail based on their interaction characteristics and associated energetics. In Mode 1 and Mode 2, strong interactions were observed. Upon adsorption, a dissociation event occurs wherein the –OH moiety from the peroxide linkage of γ-G and a proton (H) from the surface oxide group of GC combine to form a water molecule. This reaction pathway is evident in both configurations, as illustrated in Fig. 4a and b. These findings suggest a favorable proton-transfer mechanism, facilitated by the close proximity and chemical compatibility of the reactive sites on both units. In contrast, Mode 3 exhibits only weak interaction, with no evidence of bond cleavage or molecular dissociation as shown in Fig. 4c. The calculated interaction energies for the three configurations reported in Table 2 indicate that Mode 1 is the thermodynamically favorable configuration, consistent with dissociation and the formation of water.

**Fig. 4:**
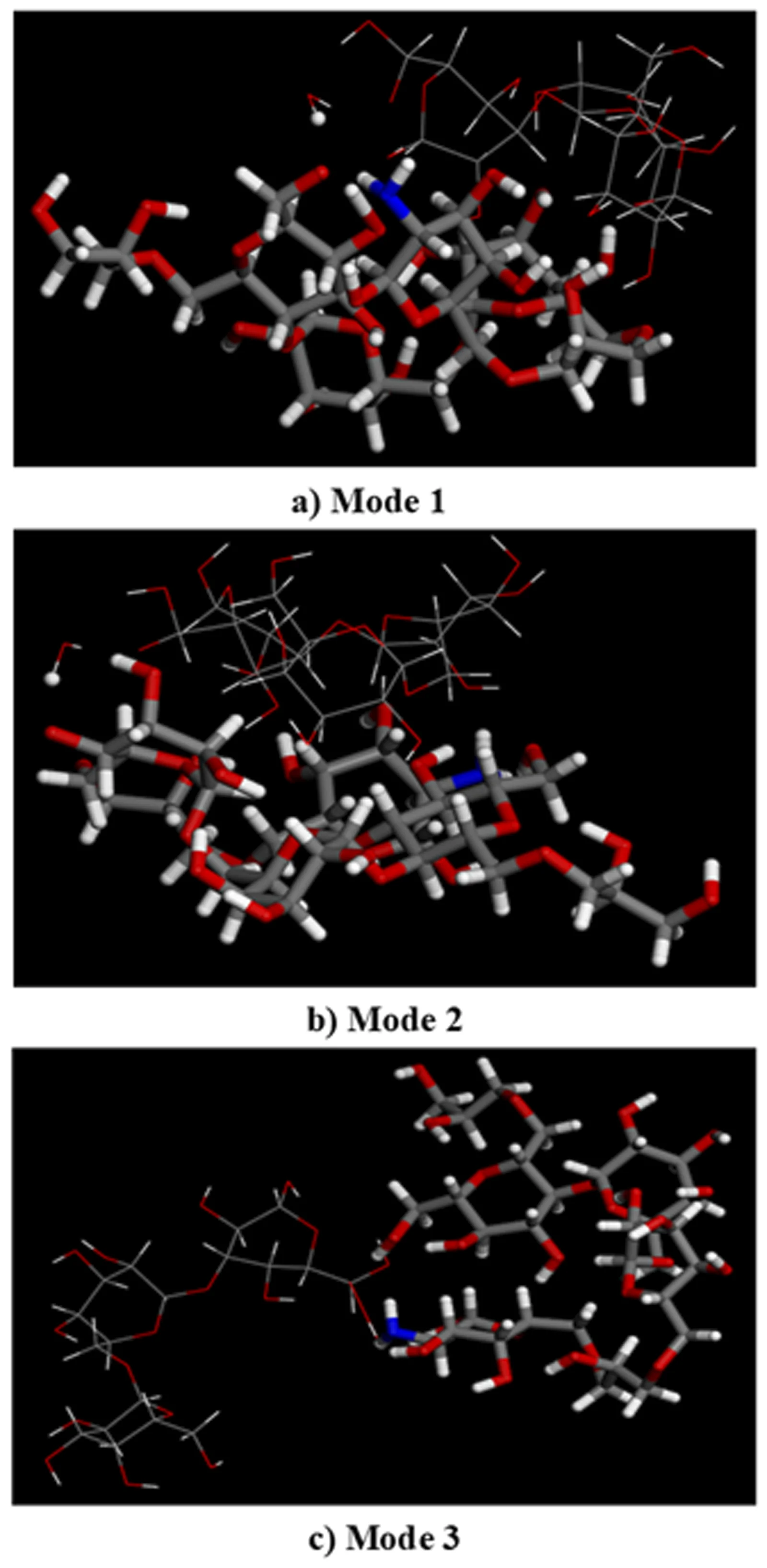
Different modes of interaction of GC and γ-G: (a) Mode 1, (b) Mode 2 and (c) Mode 3. GC has a line representation, and γ-G has a stick representation.

**Table 2:** The calculated interaction energies for the three configurations

Sr. No.	Configuration	Binding energy (eV)
1	Mode 1	−9.516
2	Mode 2	−9.214
3	Mode 3	−4.409

### 3.3. ATR-FTIR and NMR

FTIR was performed to identify the functional groups present in the samples and to characterize the crosslinking behavior, thereby aiding in proposing Scheme 1. The spectral analysis of pure *guar gum*, γ-irradiated *guar gum* (6 kGy) and chitosan was discussed in the SI (Fig. S1). The alteration of the vibration spectrum of the hydrogel films with varying concentration of the crosslinker was observed in Fig. 5a. The broadband, along with a slight shift in the range from 3000–3500 cm^−1^, indicated an intermolecular hydrogen-bonded O–H peak between *guar gum*, chitosan, and glycerol as per the work done by M.S. Rao *et al.*^67^ It was observed that GC alone showed hydrogen bonding between *guar gum*, chitosan, and glycerol, giving a broad peak at 3276 cm^−1^. Still, incorporation of γ-G increased the peak intensity while shifting its location slightly to 3272 cm^−1^, indicating that the crosslinker induced intermolecular hydrogen bonding. 2921–2885 cm^−1^ corresponded to the asymmetric and symmetric stretch of the CH_2_ stretch, where a prominent difference was observed in crosslinked GC-γ-G due to crosslinking *via* the C6 CH_2_OH group. The peak at 1020 cm^−1^ was due to the C–O–C bond, where its high intensity in GCγ was not only due to the pyranose ring of *guar gum* and chitosan but also due to crosslinking through this chemical bond formation at C6 and C4 positions (Scheme 1).^68^ A sharp, prominent peak at 1500 cm^−1^ and 1400 cm^−1^ confirmed the presence of *cis*-amide II groups of chitosan, which conferred the incorporation of chitosan into the polymer matrix.^69,70^ The weak peak at 1640 cm^−1^ confirmed the bending vibrations of –OH groups in both chitosan and *guar gum*. 924 cm^−1^ gives the *N*–H bending frequency, and the peak around 860 cm^−1^ confirms the C–H deformation caused by the ring strain.^41^ The quantitative FTIR analysis to determine the variation in the C–O–C bond has been depicted in SI Table S1 where the normalized value in GCγ-400 (1.77) compared to the other formulations where GCγ-200 (1.16) showed the lowest amongst the formulations.

**Fig. 5:**
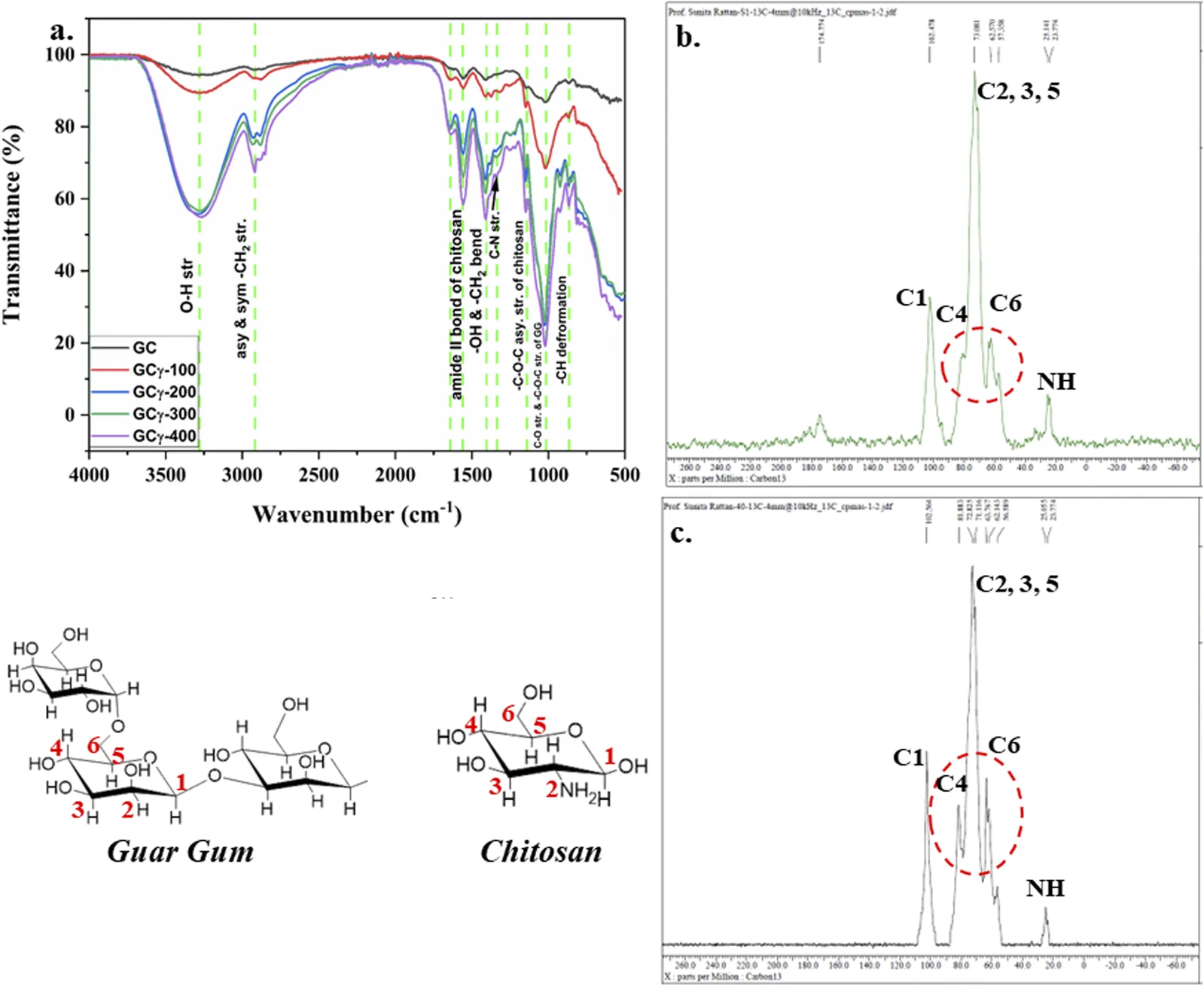
(a) FTIR spectra of hydrogel in the absence of crosslinker (GC) and in the presence of crosslinker in varying concentration (GCγ-100, GCγ-200, GCγ-300 and GCγ-400). (b) NMR spectra of GC showing no peak at C4 and no distant peak at C6, (c) NMR spectra of crosslinked GC*γ* showing crosslink formation in the C4 and C6 positions.

Scheme 1 was further supported by ^13^C solid–state NMR analysis to determine the crosslinking position in the material. A. Neppel *et al.* stated that on addition of the crosslinker, not only new resonance peaks are observed in the solid-state NMR spectra, but also with a change in the carbon environment, the carbon peaks showed either upshift or downshift along with doublet formation.^71^ As both *guar gum* and chitosan are polysaccharides, the peaks in the carbon environment were quite similar, as supported by A. D. Štiglic *et al.*^72^ On observing the NMR spectra of both GC (Fig. 5b) and GCγ (Fig. 5c), a clear distinction was observed in the C6 and C4 positions of the polysaccharide unit. No prominent peak was observed at the C4 position of the mannose unit in *guar gum* by GC. Still, on addition of the crosslinker, the singlet at 81.8 ppm was not only suggestive of the formation of a new bond but also indicated the presence of oxygen in its vicinity. Apart from that the peak at 62.5 ppm in GC was due to the CH_2_OH group of *guar gum* and chitosan at C6 position, but a doublet was formed with an upfield shift at 63.7 due to the addition of γ-G in GC inferred successful C–O–C bond (63.7 ppm) formation between GC and γ-G at C6 position of mannose unit and 62.14 ppm depicted the intact CH_2_OH groups in C6 position of galactose unit of *guar gum* as suggested in Scheme 2. The prominent overlapped peaks of C2, 3, and 5 carbon atoms showed a slight downshift and a slight doublet formation from 73 ppm to 72.8 and 71.116 ppm, due to changes in the oxygen environment resulting from the addition of γ-G, which contained peroxides formed after γ irradiation. The characteristic doublet peak of chitosan due to the CH_2_NH_2_ bond was observed at 25 and 23 ppm, and the doublet was attributed to the NH_2_ group, another prominent binding site, which crosslinked with γ-G *via* the glycerol bridges.

### 3.4. Swelling studies and swelling kinetics of the hydrogels

To fabricate a stimulus-responsive hydrogel, the swelling behavior of the prepared hydrogel films was analyzed under different solvent conditions. This behavior differed depending on the type of modification, *e.g.*, crosslinking density is inversely proportional to the swelling ratio.^73^ Crosslinking also affected the pH-responsive behavior by controlling swelling and mechanical properties; excessive crosslinking reduced pH sensitivity, whereas insufficient crosslinking increased it, underscoring the need for optimal crosslinking. J. Miras *et al.* studied the pH-responsive behavior of genipin-crosslinked chitosan, where it swelled at pH 3 but shrank at pH 6 by observing the AFM and elastic modulus of the material.^74^ In addition to crosslinking density, the type of functional group also determines pH-responsive behavior. Review done by D. Mudgil *et al.* stated that *guar gum*, although it showed high stability from pH 4–7, showed rapid degradation in extreme conditions (high and low pH).^31^ This was because *guar gum* tends to show high-level hydrogen bonding because of its excessive OH groups, which allowed the hydroxyl ions of the basic medium to open these polymer chains due to electrostatic repulsions between like groups, leading to increased hydration in the basic medium.^1^ Whereas in the case of highly acidic conditions, the proton attacked the oxygen atom, leading to depolymerization of the material, but at pH 4–6, *guar gum* showed similar hydration and swelling behavior as in the case of neutral medium, owing to the stability of the material.^75^

At first the swelling behavior of different hydrogels with varying dosage (kGy) of the crosslinker (4 kGy, 6 kGy & 8 kGy) was studied out of which hydrogel having 6 kGy crosslinker showed controlled results compared to the other films which was due to C˙ radical formation at different positions in *guar gum* that depended on the dosage^24^ (Fig. 6a). The controlled swelling in the 6 kGy-irradiated crosslinker was due to modifications at sterically unhindered sites (C6 and C4 positions), which formed effective crosslinked sites. Therefore, the 6 kGy γ-G was then added in varying concentrations to obtain formulations of GC–(100–400) γ-G. The swelling patterns of these formulations were first analyzed in aqueous media, where the addition of γ-G decreased swelling, thereby helping select the lowest and highest concentrations (GCγ-100 and GCγ-400, respectively) for pH-responsive behavior (Fig. 6b). The selected hydrogel formulations GCγ-100 and GCγ-400 were compared with GC swelling behavior under aqueous and various pH conditions. GC showed maximum equilibrium swelling (300%) which was consistent with its native, non-crosslinked, hydrogen bonded network that minimized its water uptake. But the addition of γ-G decreased the swelling ratio compared to GC which differed non-monotonically in γ-G containing formulations as a function of crosslinking density, pore morphology and surface hydrophilicity where the independently established sections depicted that the swelling behavior was governed by the combined influence of these factors rather than crosslinking alone. On comparing the swelling ratios of GCγ-100 and GCγ-400, GCγ-400 showed a greater swelling ratio than GCγ-100 under both aqueous and pH conditions. This was due to radical recombination in GCγ-400 as it crossed the critical concentration that created interlinked pore morphology that is discussed in detail in Section 3.5, which increased surface area and pore volume that provided greater accessible pathways for the water uptake ones the initial Cassie–Baxter (Section 3.5) related restriction of water ingress was overcome at longer immersion time. Although GCγ-100 showed the lowest equilibrium swelling in aqueous medium, the divergence between its restricted swelling and mechanical properties that is discussed in Section 3.6 along with the lowest diffusion exponent indicated that the rapid initial uptake was due to the primary crosslinking nodes formed during the early stages of network reorganization that restricted the long-term network expansion despite not conferring substantial mechanical reinforcement. On the other hand, GCγ-200 and GCγ-400 showed comparable high equilibrium swelling relative to GCγ-100 and GCγ-300 where GCγ-200 showed surface hydrophilicity due to the increase in crosslinking activity that was also observed in its swelling kinetics and wetting analysis. GCγ-300 on the other hand showed intermediate swelling equilibrium due to its compact R–R bond that not only restricted the water uptake due to low porosity network observed in BET, but its Wenzel type of surface hydrophilicity also allowed swelling to proceed in a degree more than GCγ-100.

**Fig. 6:**
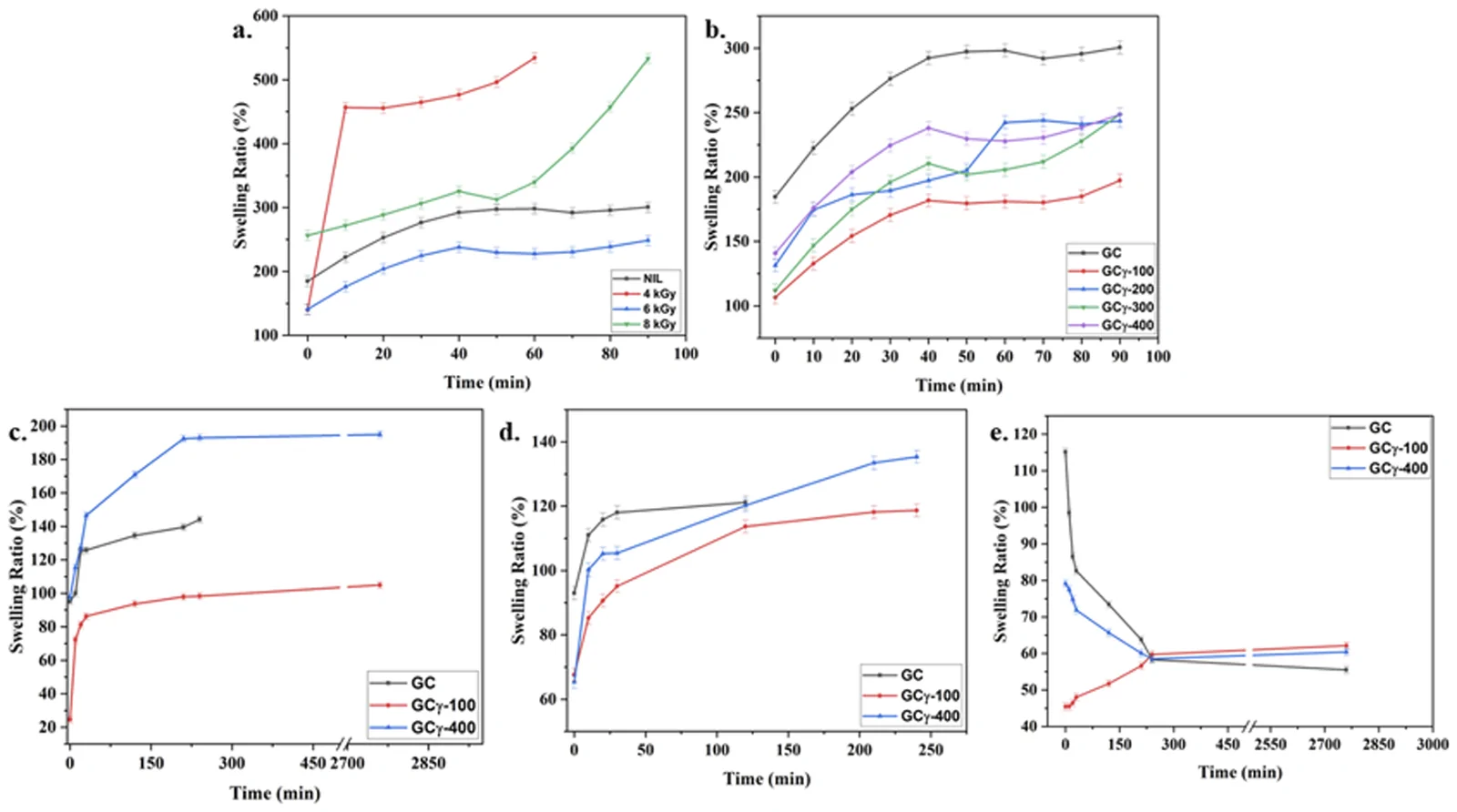
Swelling analysis of hydrogels (a) varying irradiation doze, (b) varying concentration of 6 kGy irradiated crosslinker, (c) stimulus responsive behavior of hydrogel films in pH 4, (d) stimulus responsive behavior of hydrogel films in pH 7.5, (e) stimulus responsive behavior of hydrogel films in pH 9.

To understand the pH responsive behavior, swelling ratio was observed at pH 4, 7.5 and 9 for GC, GCγ-100 and GCγ-400 (Fig. 6c–e). In acidic medium (pH 4) the swelling ratio of both the formulations increased drastically, although GCγ-400 showed maximum swelling of 195% and GCγ-100 gave the maximum value of 105% compared to the non-crosslinked, hydrogen bonded GC matrix, which is due to the additional protonation of NH_2_ group of chitosan, that generated strong electrostatic repulsions between the polymer chains to facilitate counter-ion influx that promotes both chain separation and enhanced water uptake.^43,76,77^ Although GC's swelling profile in pH 4 disrupted due to the protonated environment that led to progressive structural erosion and loss of film integrity, the flexible R–O–R glycerol bridge crosslink architecture formed *via* radical recombination in GCγ-400 allowed greater chain mobility and network expansion in response to protonation driven repulsion which is also supported by the increased pore morphology seen in BET. Whereas, GCγ-100 depicted more restricted response that described its sparse, early-stage primary crosslink nodes formed which despite being less mechanically effective still constrained the network expansion under the same repulsive forces. In neutral conditions (pH 7.5), the swelling ratios were lower than that of the acidic medium with GC, GCγ-100 and GCγ-400 reaching the values of around 120% in GC and GCγ-100 but, relatively higher in GCγ-400 (around 130%). This is because in neutral conditions, the reduced electrostatic repulsions allowed increased intra and interchain hydrogen bonding which limited the water uptake relative to the fully protonated acidic state. Although the electrostatic repulsions also decreased in GCγ-400, the greater conformational glycerol bridge crosslinks lead to network expansion in pH 7.5 conditions. In basic medium (pH 9), GC showed the highest initial swelling before declining sharply at longer immersion time (55–60%) and, GCγ-400 showed comparatively lower initial swelling that gradually decreased to similar equilibrium values showing the trend similar to GC, whereas GCγ-100 depicted an opposite trend where it started from lower initial swelling and gradually increased with all 3 formulations showing similar equilibrium swelling ratios when immersed in pH 9 for an extended amount of time. This was because in basic medium, the excess OH environment promoted extensive hydrogen bonding that contracted the polymer matrix which favors compact lower swelling structure. Therefore, in GC the higher swelling then relatively decreased due to its native, non-crosslinked network that was more susceptible to hydrogen bonding reorganization, whereas in GCγ-400, the flexible glycerol bridged crosslinks constrained these contractions more effectively than the sparse network matrix of GCγ-100 resulting in a comparatively steady swelling equilibrium in GCγ-400. In contrast, GCγ-100 offered minimal initial resistance to hydrogen bonding driven contraction because of its sparse early stage crosslink formation which allowed it to gradually swell towards equilibrium as its matrix slowly accommodated water uptake overtime. This swelling behavior of *guar gum*–chitosan based systems has been similarly reported by Ghauri *et al.* where the system has reported to increase or stabilize under high pH conditions following an initial adjustment period.^58^ On fitting the swelling data of aqueous system to the Ritger–Peppas equation, the diffusion parameters obtained (Table 3), indicated that all hydrogel samples followed quasi-Fickian diffusion.

**Table 3:** Diffusion parameters obtained from Ritger–Peppa's equation to determine the type of diffusion followed by the hydrogel films stating that the hydrogel formulations followed quasi-Fickian diffusion

	GC	GCγ-100	GCγ-200	GCγ-300	GCγ-400
*k*	0.6098 ± 0.05	0.7304 ± 0.01	0.5514 ± 0.06	0.3282 ± 0.1	0.5014 ± 0.07
*n*	0.074 ± 0.01	0.0392 ± 0.002	0.10753 ± 0.02	0.1874 ± 0.03	0.1042 ± 0.01

The diffusion parameters varied systematically with γ-G content due to the network rearrangement where all the formulations showed quasi-Fickian diffusion which was well related to the work done by Ghauri *et al.* who reported the diffusion behavior of chitosan/*guar gum*/PVP hydrogel films.^58^ GCγ-100 showed the least *n* value (*n* = 0.0392 ± 0.002) and highest *k* value (0.7304 ± 0.01) signifying that the lightly crosslinked network gave minimal resistance to solvent ingress that dominated the rapid, diffusion controlled water uptake. Although there was decrease in *n* value of GCγ-100, the addition of γ-G in GCγ-200 showed intermediate diffusion behavior (*n* = 0.10753 ± 0.02, *k* = 0.5514 ± 0.06) where it showed lower initial uptake rate but a slightly higher diffusion exponent which is supported by the highest surface hydrophilicity observed in the contact angle and the colorimetric analysis where the increased crosslinking activity competed with the glycerol for reactive sites which displaced it from the bulk matrix which moderated the rate and extent of water uptake compared to GCγ-100 without creating compaction seen in GCγ-300. On the other hand, GCγ-300 exhibited the highest *n* (0.1874 ± 0.03) and lowest *k* values (0.3282 ± 0.1) which was due to the severe chain entanglement because of the R–R dominating crosslinks explained earlier which is visible in the pore collapse with the lowest surface area and total pore volume in BET analysis (Table 4) which is comparable to the work reported by Drápalová *et al.* where it was observed that with increasing crosslinking density or hydrogen bonding the transport number decreased in polysaccharide–based hydrogel films.^78^ In the case of GCγ-400 the *n* (0.1042 ± 0.01) and *k* (0.5014 ± 0.07) values were intermediate despite showing the highest porosity in BET due to flexible glycerol-bridged crosslinking sites (R–O–R) formed distributed, interconnected pores rather than a rapid, burst type of hydration profile. This increased pore volume coincided with the Cassie–Baxter effect observed in contact angle wetting analysis discussed in the next section where air entrapment limits the initial water uptake. This combination sustained the quasi-Fickian diffusion process despite having a porous profile.^79^ Although the *n* values in GC (0.074 ± 0.01) and GCγ-100 were below the range typically reported for polysaccharide systems it remained consistent with the quasi-Fickian behavior that was well observed in other crosslinked formulations where restricted swelling under crosslinking conditions resulted in lower solvent penetration rate compared to the polymer relaxation rate.^80^ Additionally, such low values are consistent with the original Ritger–Peppas framework where heterogeneous, multi-component or polydisperse systems (*i.e.*, *guar gum*, chitosan and glycerol in this case) showed diffusion exponents below the values observed in single component, homogenous matrices.^81,82^ The first- and second-order reactions were analyzed for the swelling samples under various pH conditions, and it was observed that all the hydrogels exhibited second-order kinetics, as the *R*^2^ values were higher than those for the first-order reaction (Fig. 7 and SI Table S2).

**Table 4:** Comparison between the pore dimensions of GC, GCγ-100, GCγ-200, GCγ-300 and GCγ-400, where the addition of γ-G in varying concentration differed the pore morphology of formulations

Polymer	Specific surface area (m^2^ g^−1^)		Total pore volume (cm^3^ g^−1^)		Average pore radius size (nm)	
	Adsorption	Desorption	Adsorption	Desorption	Adsorption	Desorption
GC	15.935	24.010	0.024	0.027	1.7063	1.5248
GCγ-100	13.39	12.32	0.021	0.025	1.9401	1.6865
GCγ-200	16.87	12.72	0.023	0.024	1.727	1.686
GCγ-300	8.31	7.51	0.015	0.018	2.179	1.687
GCγ-400	33.737	44.338	0.049	0.050	1.7061	1.5247

**Fig. 7:**
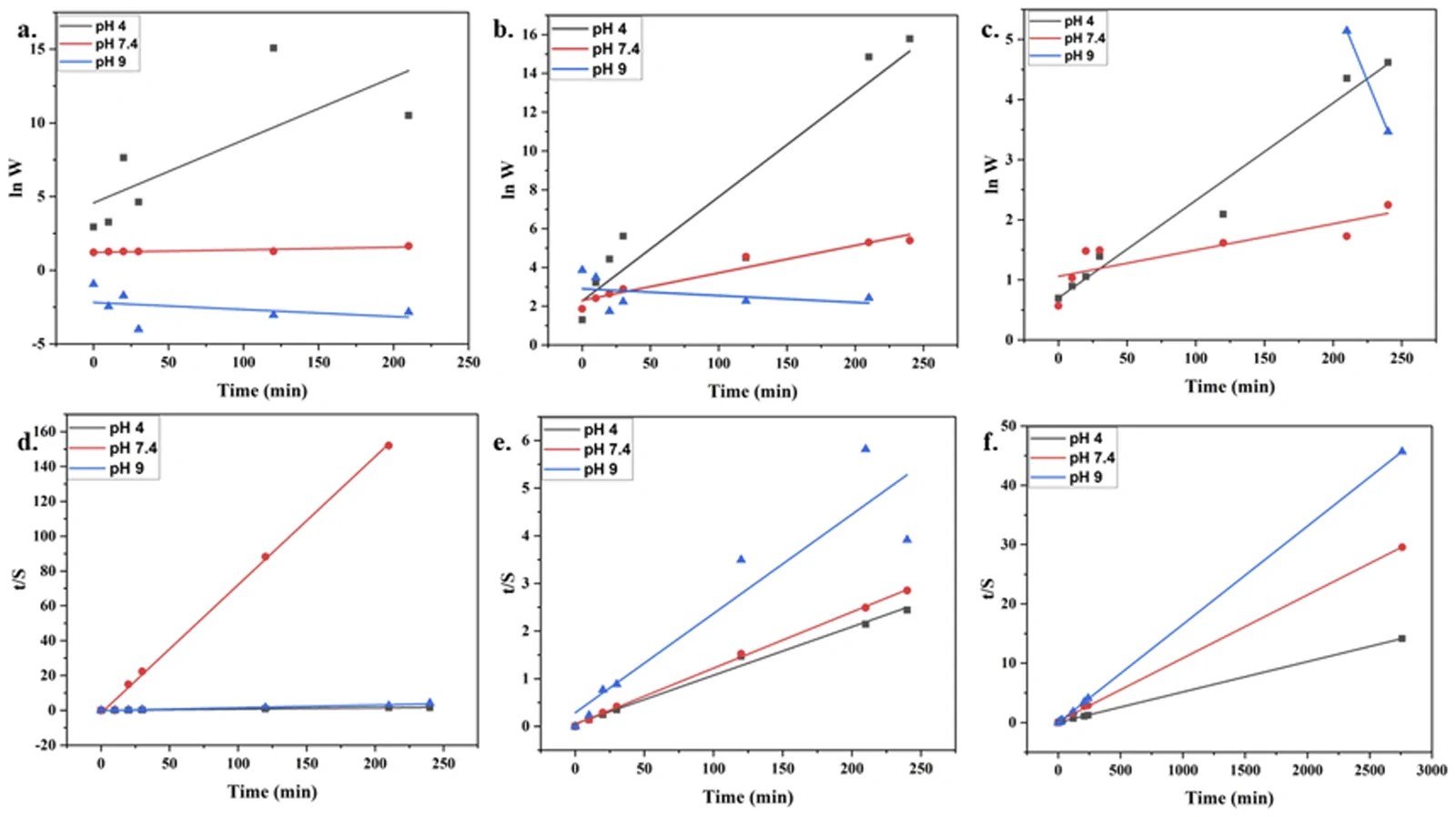
1st and 2nd order kinetics of the hydrogel films in various pH conditions. 1st order graphs of (a) GC, (b) GCγ-100, and (c) GCγ-400. 2nd order graphs of (d) GC, (e) GCγ-100, and (f) GCγ-400.

### 3.5. FESEM, contact angle and BET

The surface morphology of the crosslinked polymer matrices was thoroughly studied by Peng *et al.*, where it was observed that crosslinking smoothened the surface of the polymer particles, which varied depending on both addition and crosslinking time.^83^ On the other hand, M. Guvendiren *et al.* reported the surface patterns of crosslinked hydrogels in both dry and swollen states, where it was observed that gel uniformity and patterns were affected by the crosslinks formed and film thickness.^84^ On performing the field emission scanning electron microscopy (FESEM) of dry hydrogel samples, the non-crosslinked GC (Fig. 8a & b) showed a non-uniform surface, whereas the crosslinked GCγ-400 (Fig. 8c & d) showed uniformity due to the addition of irradiated crosslinker, which perfectly crosslinked the polymer chains. The SEM images of the freeze-dried samples at their equilibrium swollen state (Fig. 9) demonstrated a concentration dependent shift in their surface morphology across the formulation series. As the concentration of γ-G increased from GCγ-100 to GCγ-300, the films showed progressively a more defined fold and channel like structure that is also reported by K. Lewandowska who studied similar structural changes in the chitosan films with increasing crosslinking addition.^85^ Additionally, the increase in γ-G concentration led to increase in the compactness of the hydrogel films which is a characteristic property of the modified *guar gum* based films.^38,86^ In case of GCγ-400 (Fig. 10), fiber like bridging structures were observed at fractured region that connected adjacent void spaces which was due to the incorporation of glycerol bridges in the crosslink formation.^87^ This progression of isolated voids at low γ-G concentration to fiber like bridges in higher concentration samples depicted the consistency in formation of glycerol mediated (R–O–R) crosslinks due to radical recombination proposed across the formulation series.

**Fig. 8:**
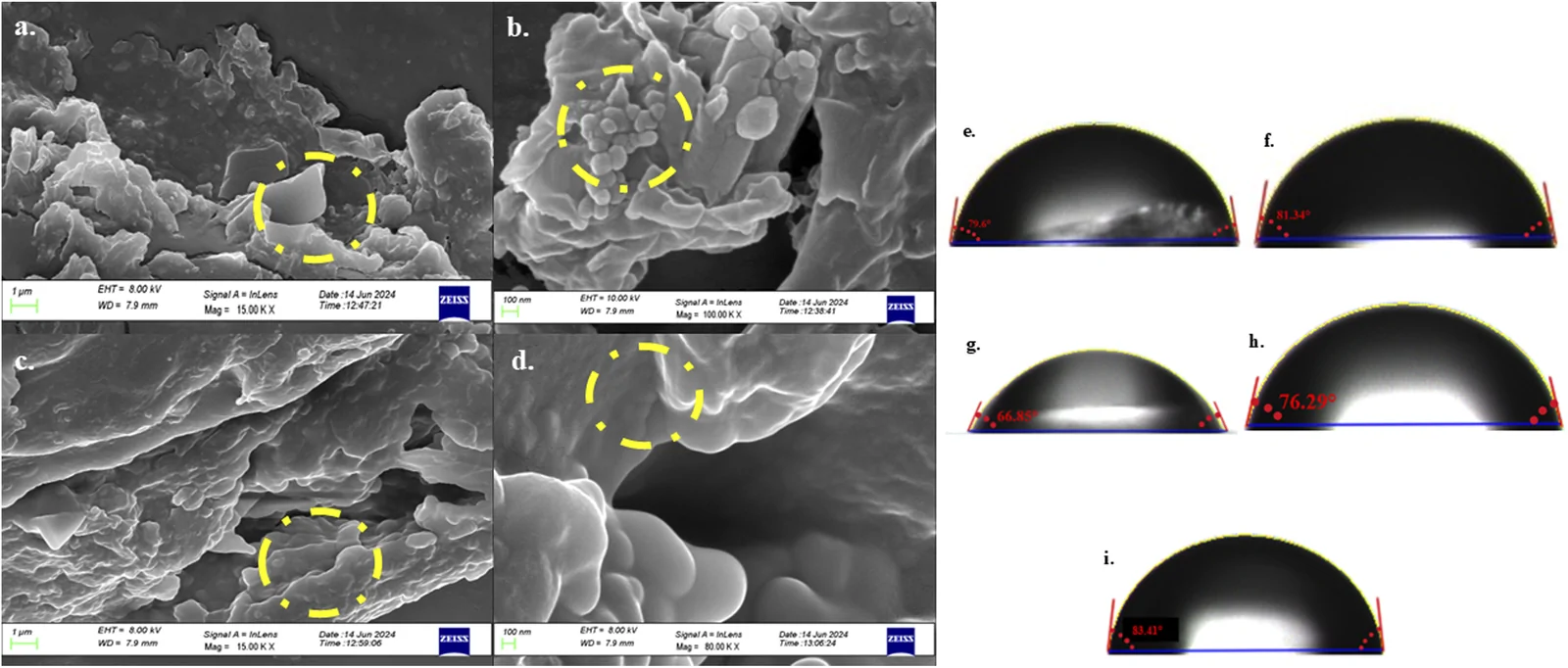
(a–d) SEM images of GC (a and b) and GCγ-400 (c and d) hydrogel films in their dry state showing surface smoothness in crosslinked film; (e–i) Contact angle of the pristine (GC: 79.6° ±3.7003) and crosslinked films (GCγ-100: 81.39° ±1.8649, GCγ-200: 66.85° ±1.1606, GCγ-300: 76.29° ±0.7995, GCγ-400: 83.41° ±1.9350).

**Fig. 9:**
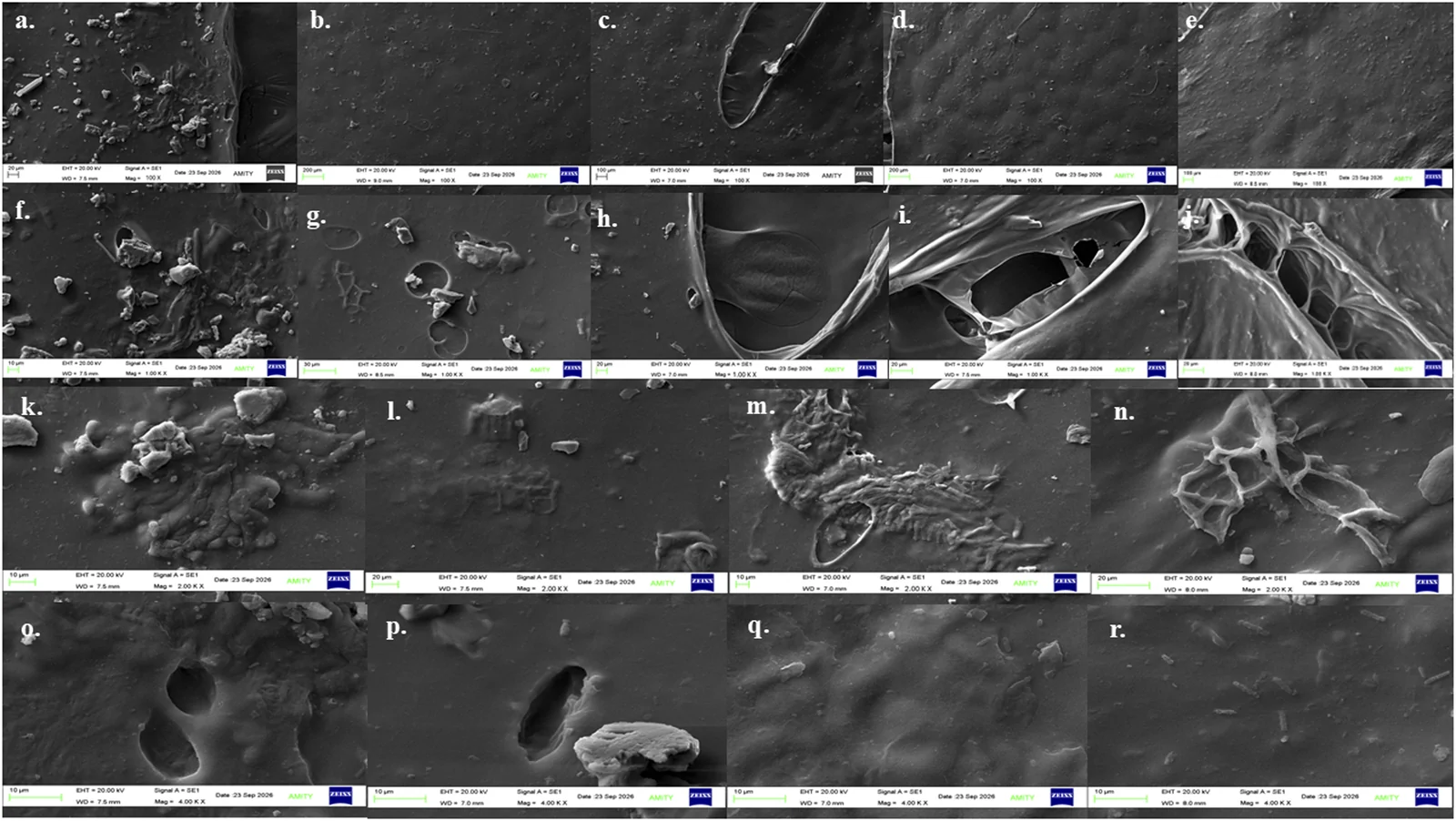
SEM images of GC, GCγ-100, GCγ-200, GCγ-300 and GCγ-400 in their swollen state at different magnifications. (a–e) Surface of the swollen hydrogel films in the order from GC to GCγ-400. (f–j) Magnified images showing the formation of fiber like bridges formed with increasing concentration where GCγ-400 (j) showed the most prominent channel like structure. (k–n) Surface fold formed in GCγ-100 to GCγ-400 due to crosslinking modification. (g–r) Increase in the compactness of hydrogel GCγ-100 to GCγ-400 films with increasing concentration of γ-G.

**Fig. 10:**
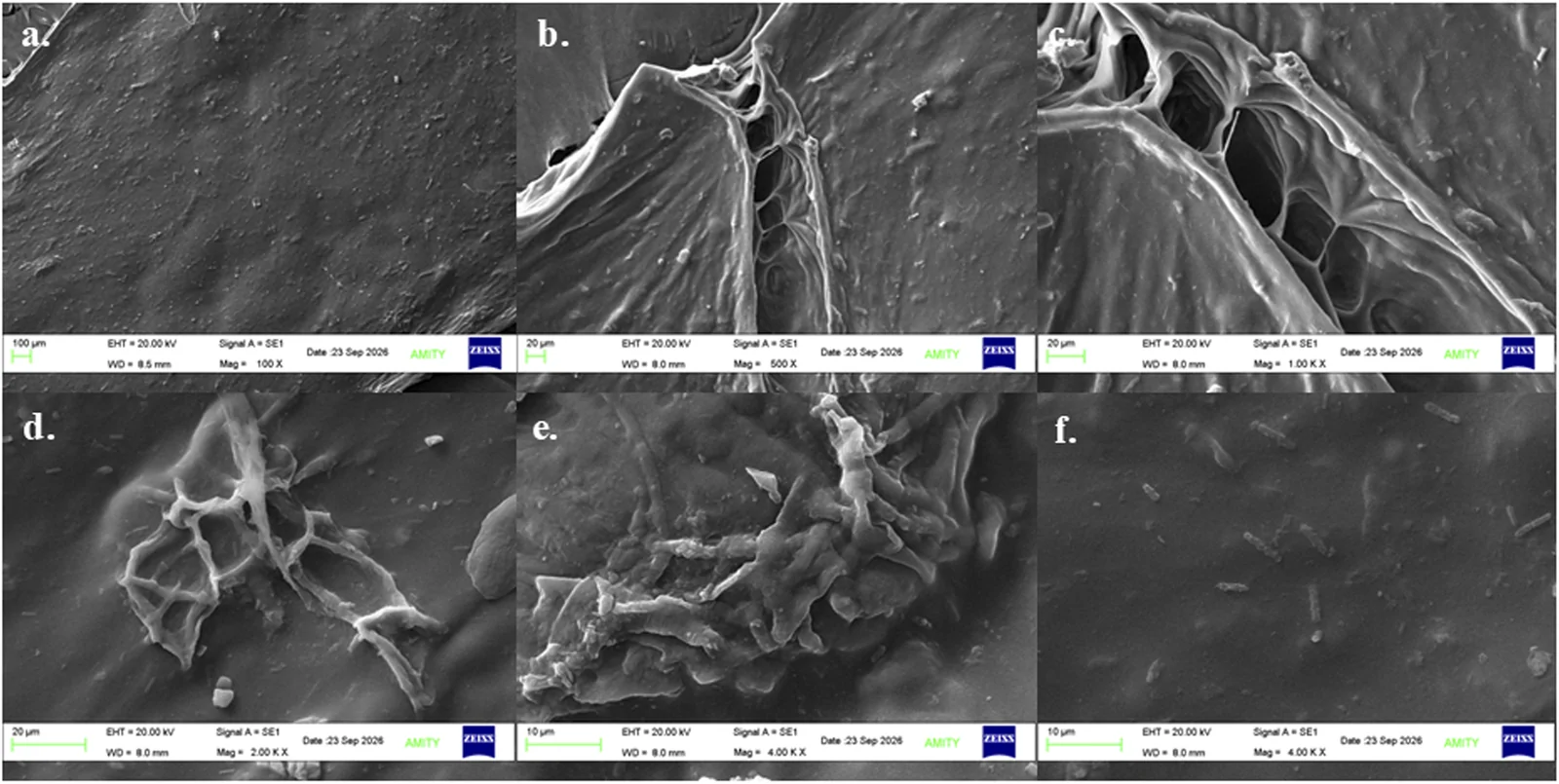
SEM images (a)–(f) of GCγ-400 at different positions in its swollen state. (a) Lowest magnified overall surface image, (b and c) fractured portion of the film, (d and e) surface foldings due to crosslinking modification observed at high resolution, (f) overall surface modification.

The properties of the crosslinker, like its functionality, length, and molecular weight, were used to determine the pore size and surface area of the hydrogel, where the crosslinker acted as a bridge that increased the distance between the polymer chains, or as a connector, where, due to multiple reactive sites, two or more polymer chains connected with each other to decrease pore size.^88^ This, in turn, also affected the surface area of the materials, which determined flexibility and other mechanical properties.^89^ This was observed in the current work, where performing the BET analysis increased both surface area and pore size. Table 4 reports the surface modifications of GC and GCγ, where γ-G altered its surface modifications. This was due to the addition of γ-G; upon irradiation, potential crosslinking sites were generated on the high-molecular-weight long-chain *guar gum* polysaccharide, making it an effective crosslinker that bridged the polymeric chains and increased the surface area of GCγ. Although the pore size of both GC and GCγ was the same nanoporous, the addition of hydrophilic crosslinker increased the pore volume, inferring that the number of pores with the same pore size (nm) was increased in the crosslinked material, which was clearly stated in Table 4. This behavior was observed only in GCγ-400 but in the case of other formulations the addition of γ-G demonstrated a non-monotonic changes in specific surface area (SSA), total pore volume and average pore radius which is consistent with network reorganization conferred in the mechanism, colorimetric analysis *etc.* which were discussed in detail in other sections.

G*uar gum* has a large number of –OH groups on its polymer chain and shows high hydrophilicity in aqueous media. To control the hydrophilic properties of *guar gum*, crosslinking was used to achieve optimal hydrophobicity, thereby controlling the hydrogel's swelling. This can be confirmed by contact angle analysis, where a higher contact angle indicates greater hydrophobicity.^38,90^ However, in the current scenario, the addition of a hydrophilic crosslinker resulted in a denser porous network, affecting the hydrogel's wetting properties. This was because it induced the Cassie–Baxter effect, which states that a layer of air trapped between the liquid droplet and the solid surface makes the hydrogel hydrophobic. But this hydrophobic nature varied with varying concentration of the crosslinker, which was attributed to the optimal number of crosslinking sites provided by γ-G. During contact angle measurements (Fig. 8e–i) to understand the wetting properties of the hydrogels, it was observed that the trend showed increasing hydrophobicity with increasing γ-G concentration (GC: 79.6° ± 3.7003, GCγ-100: 81.39° ± 1.8649, GCγ-200: 66.85° ± 1.1606, GCγ-300: 76.29° ± 0.7995, GCγ-400: 83.41° ± 1.9350). One-way ANOVA with Tukey's post-hoc comparison confirmed this difference to be statistically significant (*p* < 0.05), although the effect size is modest relative to the standard deviation of individual measurements, and some overlap in the raw data range is expected given the number of independent measurements performed (*n* ∼ 50 per formulation). This GC behavior was due to a large number of non-crosslinked free –OH groups, which induced hydrophilicity. But upon addition of γ-G at varying concentrations, the hydrogels showed variation in contact angle, attributed to the generated crosslinking sites. As GCγ-100 and GCγ-400 showed higher values, it can be inferred that sufficient crosslinking sites were available in these samples. The initial radical recombination observed in GCγ-100 results in comparatively higher contact angle due to reduction in the surface hydroxyl groups due to crosslinking. However, for GCγ-200 and GCγ-300, the hydrogels were highly hydrophilic, yielding angles of 66.85° and 76.89°. This elevated hydrophilicity in GCγ-200 because as seen earlier the reduced glycerol retention left a greater proportion of biopolymer intrinsic hydroxyl/amine groups exposed at the surface. Whereas in case of GCγ-300, showed Wenzel-type of wetting state where due to the low porous surface the densely packed surface morphology amplified the intrinsic hydrophilicity of the underlying matrix in contrast to the Cassie–Baxter state observed in GCγ-400 where trapped air increases the hydrophobicity.^91^ From solving the Cassie–Baxter equation for GCγ-400, it was observed that the solid area was 94.2% and the air area was 5.8%. This was because, on increasing the concentration of γ-G, there was an increase in the total pore volume (Table 4) of GCγ-400 due to radical recombination which formed interconnected pores, which not only affected the wettability behavior but also the mechanical properties of the hydrogel.

On observing the pore dimensions of the hydrogel formulations (Table 4), the pristine GC showed a moderate SSA and pore volume due to the native hydrogen bonded network prior to γ-G incorporation. But as the addition of γ-G was increased gradually, the pore dimensions also changed inconsistently. Relative to GC, GCγ-100 demonstrated a slight decrease in SSA whereas the adsorption pore radius increased to 1.9401 nm. This was due to the formation of primary crosslinking nodes formed where initial radical recombination reduced the number of accessible surface pores while reorganizing towards fewer, moderately larger channels which reduced the hydrogen bonding availability in the formulation which led to the slight reduction in the bound glycerol. But in the case of GCγ-200, the competing crosslinks formed that displaced glycerol from the bulk matrix showed a partial recovery in SSA and average pore radius closer to that of the native GC. On the other hand, GCγ-300 showed a more prominent decrease in both SSA and pore volume along with the largest adsorption–desorption pore radius compared to the other formulations. This is because the dominant R–R crosslinks formed in GCγ-300 showed restricted network entanglement which showed the pore dimensions similar to the ones reported by Nicu *et al.* in crosslinked polysaccharide matrices.^92^ This effect of chain entanglement due to dominant R–R formation showed ink-bottle type pore geometry where the narrow pore leads to wider internal cavity.^93^ This was also observed in other characterizations like mechanical and physically trapped glycerol in these constricted pore junctions which are discussed in other sections. In contrast to other formulations GCγ-400 showed the highest SSA and total pore volume although the average pore radius remained identical to GC indicating that the porosity increased due to increase in the number of similar sized pores throughout the matrix rather than forming one large pore. This attributed to the interconnected pore formation due radical recombination that led to glycerol-bridged crosslinking which occurred in a distributed manner throughout the network rather than localized, restricted crosslinks formed in GCγ-300. This increase in the surface area and pore density along with the increased surface hydrophobicity coincide with the Cassie–Baxter wetting behavior which explains the moderate water uptake kinetics in GCγ-400 that shows the highest porosity.

### 3.6. UTM analysis

The mechanical properties of various fabricated materials are studied using the universal testing machine to understand their applicability. In the dry state, the varying γ-G concentration in the hydrogel films affected the stress–strain relationship.^41,94^ It has been shown that, for a material to have good mechanical properties, there must be an optimal, homogeneous crosslinking network between the polymer chains.^88^ Apart from crosslink formation, pore size and distribution also play crucial roles in altering its mechanical properties. In general, an increase in the total pore size and volume reduces the Young's modulus and increases tensile strength, toughness, and, in some cases, even the elongation at break, where homogenous small pore structures further promote flexibility through uniform deformity of the polymer matrix.^95^ This general relationship, however, does not hold uniformly across the present formulation where the addition of γ-G altered the total pore volume non-monotonically across the formulations that closely tracked the crosslinking behavior established by BET analysis, rather than decreasing progressively with increasing crosslinking concentration as might otherwise be expected. Notably, the average pore radius of GCγ-400 remained comparable to GC despite its high pore volume indicating that the additional porosity arises from increase in the number of similar sized pores rather than pore enlargement. This allowed the comparable local deformation behavior between these two formulations, while the other altered crosslink types and densities arising from varying γ-G concentration drastically effected the Young's Modulus, tensile strength, toughness and elongation at break (Fig. 11a–f). The consistent shift in the dominant crosslinking architecture from rigid backbone–backbone junctions to flexible glycerol bridged (R–O–R) crosslinks of the formulations where the R–R crosslinks in GCγ-300 (Scheme 1 (i)) compacted the network and reduced the porosity and R–O–R in GCγ-400 (Scheme 1 (ii and iii)) increased the number of interconnected pores which accommodated greater into failure. This type of crosslinking transition accounts for not only increase in pore volume but also in toughness and elongation at break observed in GCγ-400 that is supported by BET, colorimetric and mechanical evidences.

**Fig. 11:**
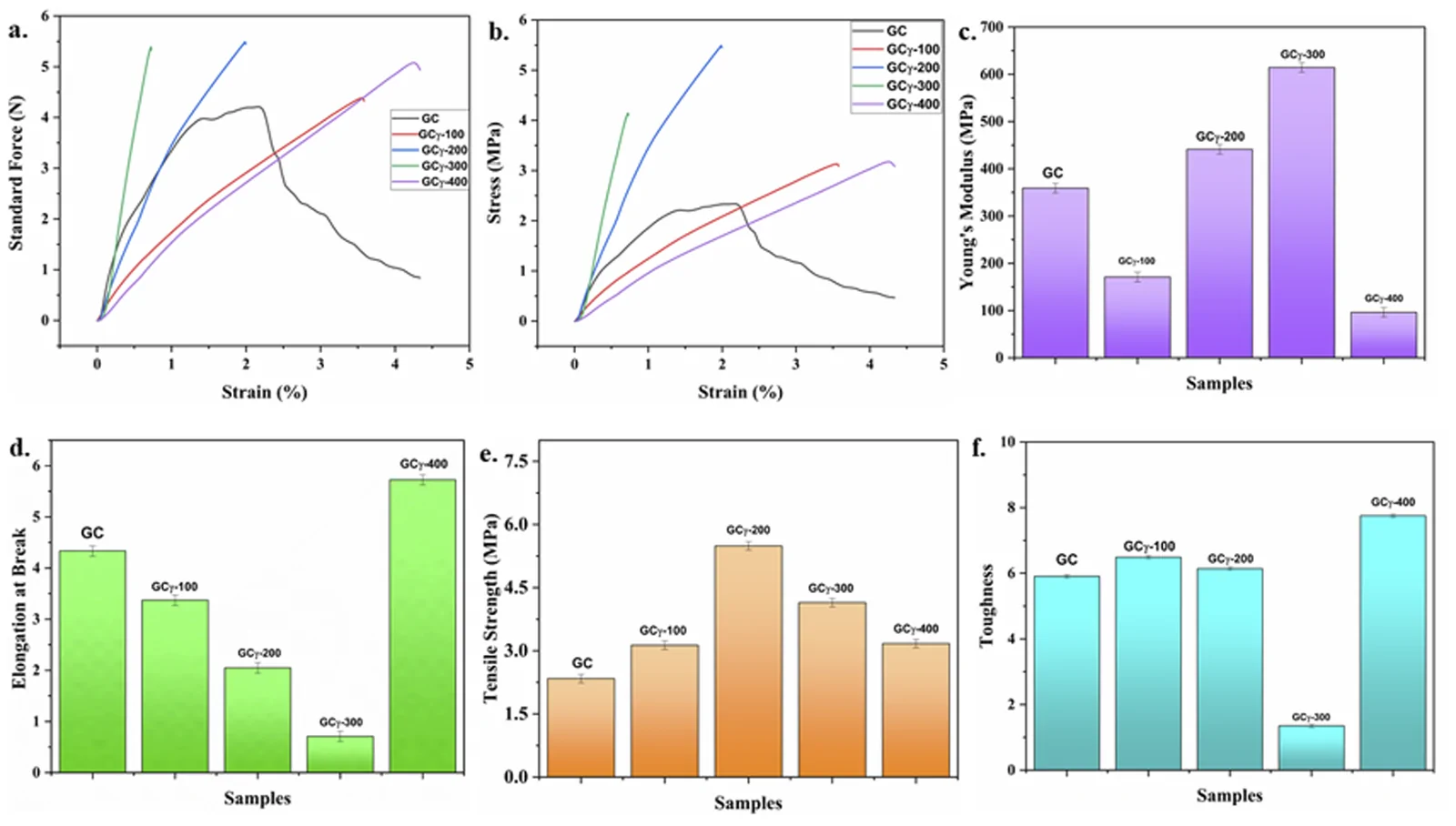
Non-monotonic variance in the mechanical properties of GC/γ-G specimens (a)-(f) due to a change in the concentration of γ-G, where GCγ-400 gave the most robust film. (a) Standard force *vs.* strain, (b) stress *vs.* strain (c) Young's Modulus (d) elongation at break, (e) tensile strength, and (f) toughness.

While looking at the stress strain graph of the formulations (Fig. 11b), it was observed that GC exhibited a moderate initial rise in stress followed by a gradual multi-step yield decline rather than an abrupt fracture due to its progressive chain slippage because of the hydrogen bonded physical network. In the case of GCγ-100, both the stress–strain curve and the force displacement curve (Fig. 11a and b) showed a shallower initial rise and a lower non-linear profile compared to GC, which lead to reduction in Young's modulus (from 359.182 to 171.034 MPa) but the toughness remained comparable to GC (5.9069 to 6.4901). This was due to the initial crosslink formation which was also seen in the reduction of bound glycerol observed in colorimetric analysis. But in the case of GCγ-200 and GCγ-300, the prior showed a sustained rise in stress reaching the highest tensile strength of (5.4904 MPa) before an abrupt fracture, while the Young's modulus (441.301 MPa), elongation at break (2.049%) and toughness (6.1424) depicted the intermediate stage of network reorganization which can be correlated with the least bound glycerol that also altered the surface hydrophilicity in GCγ-200. GCγ-300 on the other hand exhibited the steepest initial slope with highest Young's modulus (614.557 MPa) and tensile strength (4.1453 MPa) but fracturing abruptly at the lowest strain observed (0.7101%) which corresponds to its minimal toughness (1.3516). This combination of high stiffness, and strength along with minimal elongation is a characteristic of a stiff, brittle material due to their severe chain entanglement which was also observed in the collapse of the pore morphology in BET analysis. This also reflected that the rigid, backbone R–R crosslinking dominated the network formation in GCγ-300 which further supported the Wenzel-type of wetting behavior.

In contrast GCγ-400 depicted the shallowest initial slope after GCγ-100, that corresponds to the lowest Young's modulus (96.218 MPa) with the highest elongation at break (5.7246%) and toughness (7.7548) despite its tensile strength (3.1725 MPa) that is comparable to GCγ-100 rather than exceeding GCγ-200 and GCγ-300. Both the Fig. 11a and b curves show a saw tooth pattern where small partial recoveries along with small, repeated stress drops are observed before the final failure. This pattern is usually observed in networks with sacrificial or reversible crosslinks which break and reform to absorb energy before the whole network fails according to the study done by Li *et al.*^96^ This behavior was conferred on GCγ-400 because the crosslinks distributed once the concentration of γ-G crossed the critical threshold, radical recombination were feasible which allowed the incorporation of glycerol as a flexible, glycerol-bridge rather than a rigid R–R junction that dominates in GCγ-300. The combination of high elongation and toughness indicates that the flexible glycerol-bridge in GCγ-400 provided a more damage tolerant mechanical response than a rigid, brittle network formed in GCγ-300.

## 4. Future prospects and study limitations

The study was limited to the effect of pre-irradiated *guar gum* in varying concentration on the physical and chemical properties of the *guar gum*–chitosan where a detailed mechanistic insight was provided that was supported by both structural (FTIR and NMR) and *in silico* (DFT) interaction analysis, along with their hydration, swelling and mechanical properties. Although known materials were selected, the primary purpose of the study was to show that gamma irradiated biopolymers can be used as a crosslinker in their respective matrix. This methodology was chosen to overcome disadvantages like homopolymerization that are generally observed in a mutual irradiation technique. Additionally, it focused on identifying a sustainable alternative to the chemical crosslinking method by using green solvents and techniques.

Swelling and mechanical properties were studied as they are inversely and directly proportional respectively to the crosslinking ratio which are the basis for the hydrogel applicable. But, additional application-based studies like the drug kinetics and biocompatibility, water and gas permeability, dye absorption studies *etc.*, would strengthen the understanding of these properties in various applications across drug delivery systems, packaging, water purification *etc.* Therefore, in this study, optimization of γ-G clearly affected the swelling and mechanical properties by controlling the swelling ratio and enhancing mechanical properties. Not only the experimental data, but also the computational interaction studies using DFT showed that a strong interaction is possible between γ-G and GC matrix which was confirmed not only through NMR but also through strong binding energy of −9.516 eV. Although DFT interaction studies were performed to obtain the binding energy, a comprehensive reaction pathway analysis could provide deeper insights into the exact molecular dynamics. Apart from that, the average pore radii of all the formulations (1.5–2.2 nm) fall at or below 2 nm micro/mesoporous boundary, where BJH analysis becomes less accurate for absolute pore size due to the limitation of the Kelvin equation.^93^ But this does not affect the relative trend discussed above where crossing the threshold concentration of pre-irradiated material lead to radical recombination which is observed across the formulations that is supported independently by mechanical, colorimetric and swelling kinetics data. Furthermore, future studies should focus on optimizing this hydrogel framework for the practical and specific deployment in various applications.

## 5. Conclusion

The above results showed successful crosslinking of *guar gum*–chitosan hydrogel films *via* the γ-modified *guar gum* unit (γ-G), which provided potential crosslinking sites, yielding a robust, pH-responsive, nanoporous *guar gum*–chitosan hydrogel film. Structural characterizations, analyzed by FTIR, NMR, and DFT, showed that the site with the highest crosslinking potential was at the C6 and C4 positions of the mannose chain of *guar gum*, which helped predict Scheme 2. Through DFT analysis, it was also confirmed that *guar gum*, when pre-irradiated with γ rays, did create oxygen sites, which further formed a stable peroxide at the C6 position of the mannose chain in comparison to the galactose C6 position, resulting in a strong chemical bond formation with the polymer chain *via* glycerol which is incorporated as a flexible crosslinking bridge rather than solely acting as a plasticizer. Experimentally, the addition of the synthesized crosslinker in varying concentrations affected the polymer's swelling, wetting, pH responsiveness, and mechanical properties non-monotonically. While GCγ-100 showed comparatively sparse crosslink network, GCγ-200 exhibited an intermediate stage of network reorganization which increased the Young's Modulus and tensile strength along with surface hydrophilicity, GCγ-300 exhibited a rigid, brittle network arising from severe chain entanglement and pore compaction, and GCγ-400 showed the most promising overall performance owing to its dense, homogenous, flexible crosslink network. This was because of the radical induced recombination as suggested in Scheme 1 where a rigid backbone–backbone (R–R) crosslink formation dominated in GCγ-300 and flexible glycerol-bridge dominated in GCγ-400. The formation of glycerol-bridge (R–O–R) in GCγ-400 was attributed to the excess concentration of γ-G which exceeded the critical concentration that provided sufficient reactive sites that formed crosslinks with adjacent groups to form interlinked porous morphology without collapsing the original pore structure as in the case of GCγ-300. On comparing GCγ-400 with pristine GC, it was observed that the pore size (1.706 nm) remained comparable, despite its two-fold increase in the adsorption surface area from (15.935 to 33.737 m^2^ g^−1^) and a similar total pore volume increase from (0.024 to 0.049 cm^3^ g^−1^) which supported the hydrophobic behavior of the crosslinked hydrogel due to the Cassie–Baxter effect. Further support of an effectively crosslinked GCγ-400 hydrogel was provided by its pH-responsive behavior and mechanical properties, which showed that GCγ-400 displayed controlled pH responsiveness along with enhanced toughness and elongation at break. This confirmed that pre-irradiation of *guar gum* with γ irradiation can serve as a successful alternative to the chemical modifications for creating crosslinks in *guar gum*–chitosan hydrogel films, thereby fabricating a robust pH-responsive nanoporous polymer matrix with greater surface area, which can be further studied for stimulus responsive applications.

## Abbreviations

γ: Gamma
GC: *Guar gum* + Chitosan + Glycerol
γ-G: Irradiated *Guar gum*
GCγ- (100, 200, 300 and 400): Formulations with varying concentration of γ-G
C˙: Carbon free radical
COO˙: Peroxidation of carbon free radical
DFT: Density Functional Theory
DMol^3^: Density Functional Molecular Orbital version 3
DNP: Double numerical polarization
DSPP: DFT semicore pseudopotentials
GGA: Generalised gradient approximation
PBE: Perdew–Burke–Ernzerhof
BJH: Barrette–Joyner–Halenda
*E*_BE_
: Binding energy
*E*_Complex_
: Total energy of optimised complex
*E*_1_
: Total energy of GC unit
*E*_2_
: Total energy of IrGG
*δ*
: Chemical shift in ppb
*F*
: Fractional swelling
*t*
: Time
*k*
: Rate constant of swelling
*n*
: Diffusional exponent
*R* ^2^
: Coefficient of determination
*S*_∞_
: Swelling at equilibrium
*S*_*t*_
: Swelling at time *t*
*k*_1_
: First order kinetics
*k*_2_
: Second order kinetics
*θ*_*m*_
: Contact angle on solid air gaps
*θ*_*i*_
: Equilibrium contact angle
*f*_1_
: Total area of solid under the drop
*f*_2_
: Total area of air under the drop

## Author contributions

K. Dhana Sai Shree designed and conducted the experiments, analyzed the data and has written and edited the original draft after thorough investigation. Jogender carried out the computational analysis and has written after detailed analysis. Eric. W. Cochran has validated and supervised the entire study and thoroughly edited the final manuscript. Sunita Rattan has conceptualized the work and validated, supervised, and provided resources for the work and edited the manuscript.

## Conflicts of interest

The authors declare that there is no conflicts of interest.

## Supplementary Material

RA-OLF-D6RA06085D-s001

## Data Availability

The data supporting the article has been included as a part of supplementary information (SI). Supplementary information: FTIR of *guar gum*, chitosan and γ-G; Viscosity analysis of *guar gum*, chitosan and γ-G to obtain the molar mass; comparison table of 1st and 2nd order kinetics; Fickian plot; ^13^C NMR spectra of GC and GCγ; optimized labelled structure of GC and γ-G; Hirshfeld Charge and Mayer Bond order for GC and γ-G. See DOI: https://doi.org/10.1039/d6ra06085d.
