# Supplementary material for: Mechanistic insights on irradiation induced fabrication of nanoporous guar gum/chitosan pH responsive hydrogels: a combined DFT and spectroscopic study
Source: RSC Adv. 2026 Oct 20. Online ahead of print. doi: 10.1039/d6ra06085d (PMC13682629; doi:10.1039/d6ra06085d)
Supplement: Supplementary file 1 [file RA-OLF-D6RA06085D-s001.pdf]

Supplementary Information (SI) for RSC Advances.
This journal is © The Royal Society of Chemistry

Mechanistic Insights on Irradiation Induced Fabrication of Nanoporous *Guar Gum/Chitosan* pH responsive Hydrogel: A Combined DFT and Spectroscopic Study.

K. Dhana Sai Shree [a], Jogender [b], Eric W. Cochran [c], Sunita Rattan*[a]

[a] Amity Institute of Applied Sciences, Amity University Noida, U.P., 201313, India.

[b] Pradhanmantri College of Excellence SLP Government Post Graduate College, Morar, Gwalior, M.P., 474006, India.

[c] Department of Chemistry, Iowa State University, Ames, IA, 50011, United States.

* Corresponding Author email: srattan@amity.edu

Contents:

Fig. S1. FTIR spectra of **(a.)** pristine *guar gum* **(b.)** 6 kGy irradiated *guar gum* **(c.)** chitosan.

Fig. S2. Reduced Viscosity (mL/g) vs Conc. (g/mL) Graph of *guar gum*, γ-G and chitosan to obtain the intrinsic viscosity to calculate the molar mass.

Table S1. Quantitative FTIR results of the C-O-C linkage abundance across the formulation where A1020 and A1400 are the absorbance of C-O-C stretch and backbone chain reference absorbance respectively where the ratio (R) is normalized to the pristine GC value.

Table S2. Comparison table between 1st and 2nd order kinetic order of hydrogels od GC, GCγ-100 and GCγ-400 showing that highest R^2 value represents that all formulations follow 2nd order kinetics.

Fig. S3. ln F vs ln t plot of GC, GCγ-100, GCγ-200, GCγ-300 and GCγ-400 depicting that all the formulations follow Fickian type of diffusion.

Fig. S4. Microbial Degradation studies of **a**. GC, **b**. GCγ-100 and **c**. GCγ-400 in *E. Coli.*

Fig. S5. ^{13}C solid state NMR of GC

Fig. S6. ^{13}C solid state NMR of GC*γ*

Fig. S7. Optimized labelled structure of GC

Table S3. Hirshfeld Charge and Mayer Bond order for GC.

Fig. S8. Optimized labelled structure of γ-G

Table S4. Hirshfeld Charge and Mayer Bond order for γ-G.

1. FTIR spectra of precursors

FTIR analysis was performed on the precursors, *guar gum*, γ-irradiated *guar gum* and chitosan to understand the structural changes after modifying the *guar gum-chitosan* polymer chains. The characteristic structural difference between pristine *guar gum* and γ-modified guar gum (γ-G) was marked by not only shifting of peaks in the fingerprint region but also through distant change in the vibrational nature of OH, CH_2 and C-O-C groups. 3000-3500 cm^{-1} region and 1652 cm^{-1} represents the O-H bond behaviour where the broadness with increased intensity and a slight shift was due to peroxidation of C· radicals to form C-OOH when reacted with O_2 of air during γ irradiation[1]. In pristine *guar gum* the peak at 1021 cm^{-1} represents C-O-H bond which after irradiation shifted to 1005 cm^{-1} which was due to reduction in bond strength in C-OOH[2]. The prominent peak at 1156 cm^{-1} in γ-G was due to the asymmetric stretching of C-O-C bond indicating the plausible peroxide formation at C4 and C6 positions[3]. Apart from pristine *guar gum* and γ-G, pristine chitosan vibrational spectra was also studied by comparing to the already existing literature in order to understand the behaviour of the crosslinked hydrogel material. The characteristic peaks of chitosan in the region from 1380 – 1657 cm^{-1} corresponds to the free NH_2 group which later show significant changes in the crosslinked material to give their characteristic peaks as amide I and amide II bonds[4].

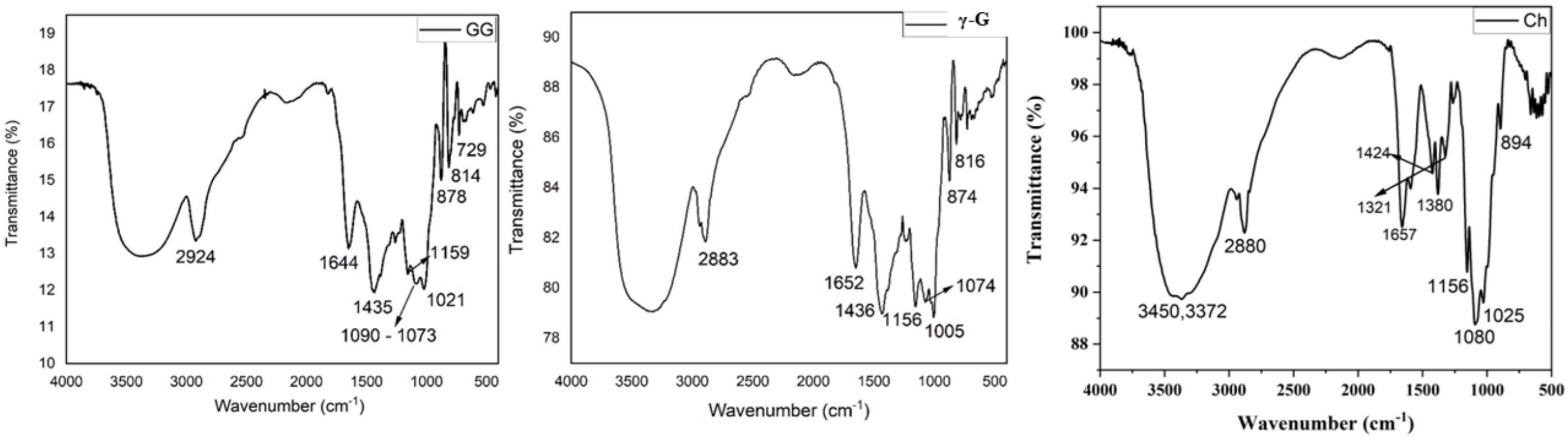

Fig. S1. FTIR spectra of (a.) pristine *guar gum* (b.) 6 kGy irradiated *guar gum* (c.) chitosan.

2. Viscometric analysis of *Guar gum*, Chitosan and γ-G

Molar masses of γ-G, *guar gum* and chitosan were obtained using the Mark–Houwink equation. To solve the equation, viscometric analysis was done using Ubbelohde viscometer at room temperature where the intrinsic viscosity was calculated from the below equation by using the Mark–Houwink constants of the respective materials obtained from literature,

$$[\eta] = KM_r^a \tag{1}$$

Where η is the intrinsic viscosity with K = 3.7×10^{-4} and a = 0.74 for *guar gum* and K = 3.5×10^{-4} and a = 0.79 for chitosan , respectively[5,6].

On analyzing the behaviour of chitosan and *guar gum*, it was observed that chitosan showed more viscous behavior in comparison to *guar gum* because of the formation of cationic site that lead to chain expansion in acidic medium whereas *guar gum* was comparatively less viscous as it remained neutral in aqueous medium[7,8]. Further, it was also observed that γ-G showed reduced molar mass and viscosity in comparison to *guar gum* due to the radiation induced chain session in the polymer chains even though it did not show drastic change in the gross confirmation[9,10]. On calculating the molar mass, *guar gum* gave the value of 91.575 ×10^{7} g/mol whereas gamma irradiation reduced the molar mass to 10.119 ×10^{7} g/mol in γ-G and chitosan gave the value of 35.627×10^{7} g/mol.

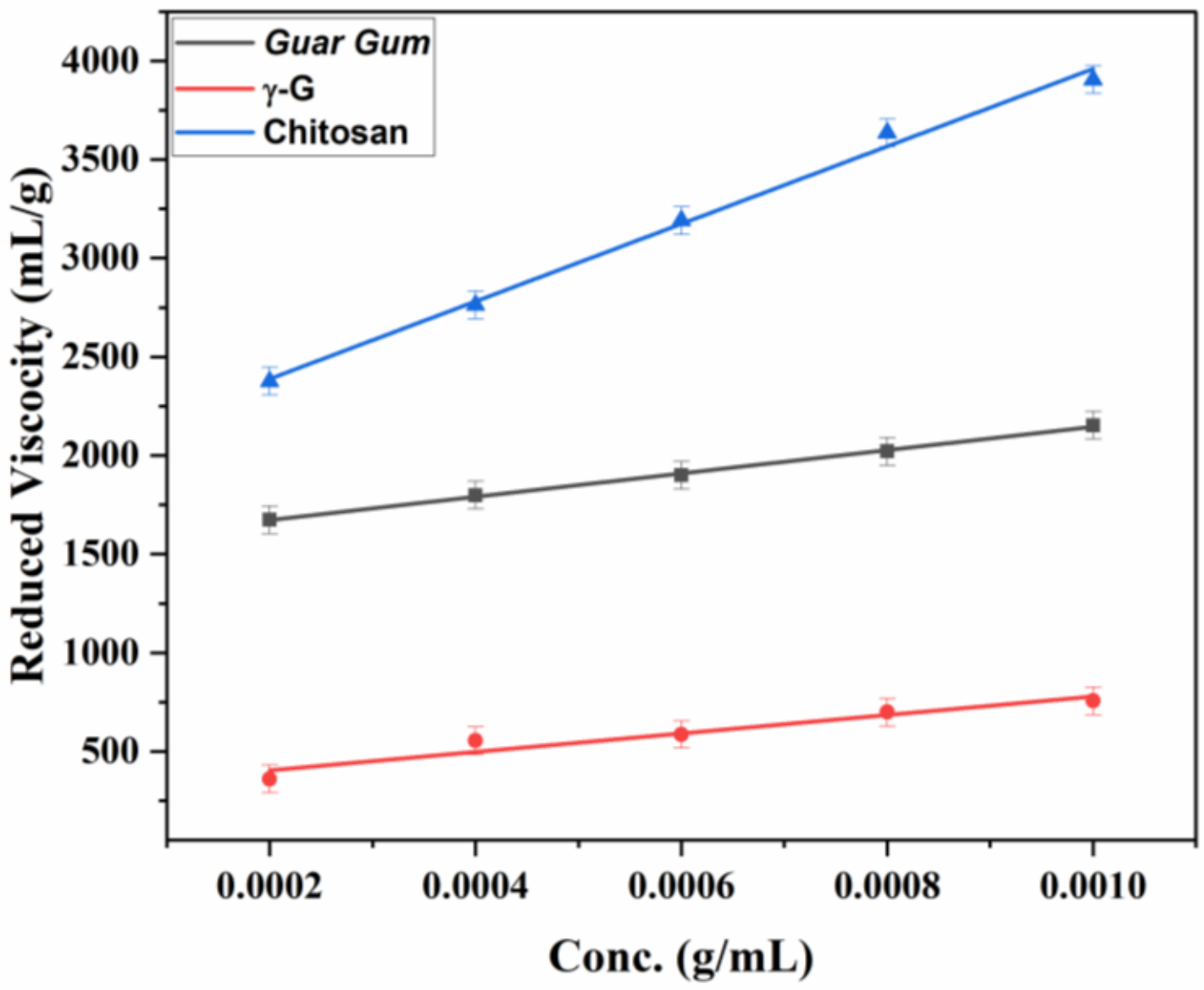

Fig. S2. Reduced Viscosity (mL/g) vs Conc. (g/mL) graph of G*uar Gum*, γ-G and Chitosan to obtain the intrinsic viscosity to calculate the molar mass.

Table S1 Quantitative FTIR results of the C-O-C linkage abundance across the formulation where A1020 and A1400 are the absorbance of C-O-C stretch and backbone chain reference absorbance respectively where the ratio (R) is normalized to the pristine GC value.

	A_{1020}	A_{1400}	R= A_{1020}/ A_{1400}	R/R_{GC}
GC	0.062	0.03232	1.91832	1
GCγ-100	0.163	0.054	3.0185	1.5735
GCγ-200	0.58847	0.2649	2.22147	1.15802
GCγ-300	0.603	0.18376	3.28145	1.71058
GCγ-400	0.7176	0.2109	3.40256	1.7737

Table S2. Comparison table between 1st and 2nd order kinetic order of hydrogels od GC, GCγ-100 and GCγ-400 showing that highest R^2 value represents that all formulations follow 2nd order kinetics.

	**GC**				**GCγ-100**				**GCγ-400**			
	**1st order**		**2nd order**		**1st order**		**2nd order**		**1st order**		**2nd order**	
**pH**	**R^2**	**k (min^{-1})**	**R^2**	**k /g_{gel} (g_{water}min)$^{-1}$**	**R^2**	**k (min^{-1})**	**R^2**	**k /g_{gel} (g_{water}min)$^{-1}$**	**R^2**	**k (min^{-1})**	**R^2**	**k /g_{gel} (g_{water}min)$^{-1}$**
**4**	0.556	0.0427	0.995	0.00189	0.875	0.0537	0.993	0.01017	0.975	0.01622	0.99997	0.00069
**7.4**	0.812	0.0017	0.998	0.75590	0.952	0.014	0.999	0.00334	0.685	0.00437	0.999	0.00059
**9**	0.138	-0.0047	0.962	0.0158	0.128	-0.0035	0.872	0.00152	1	-0.0559	0.999	-0.0092

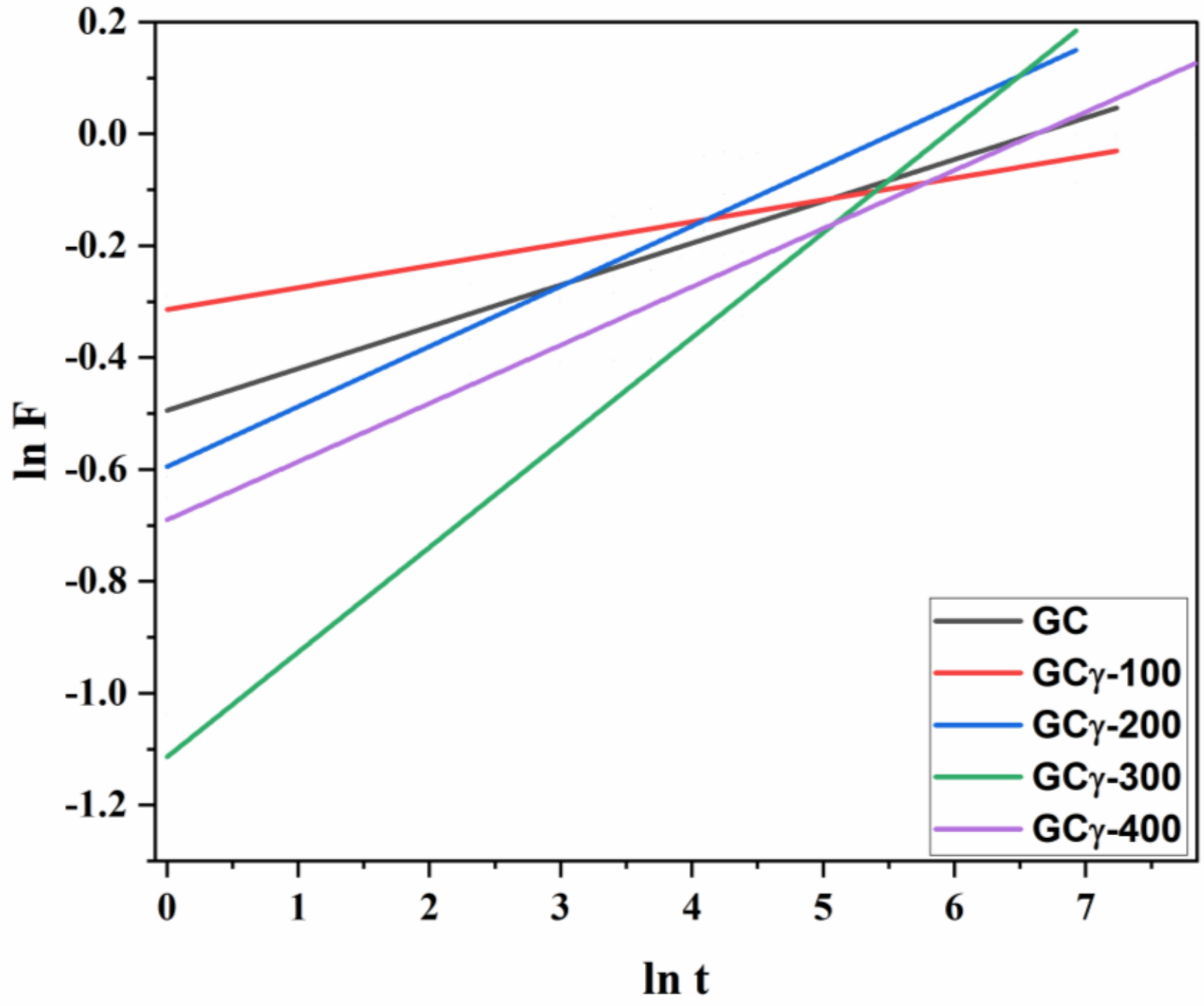

Fig. S3. ln F vs ln t plot of GC, GCγ-100, GCγ-200, GCγ-300 and GCγ-400 depicting that all the formulations follow Fickian type of diffusion.

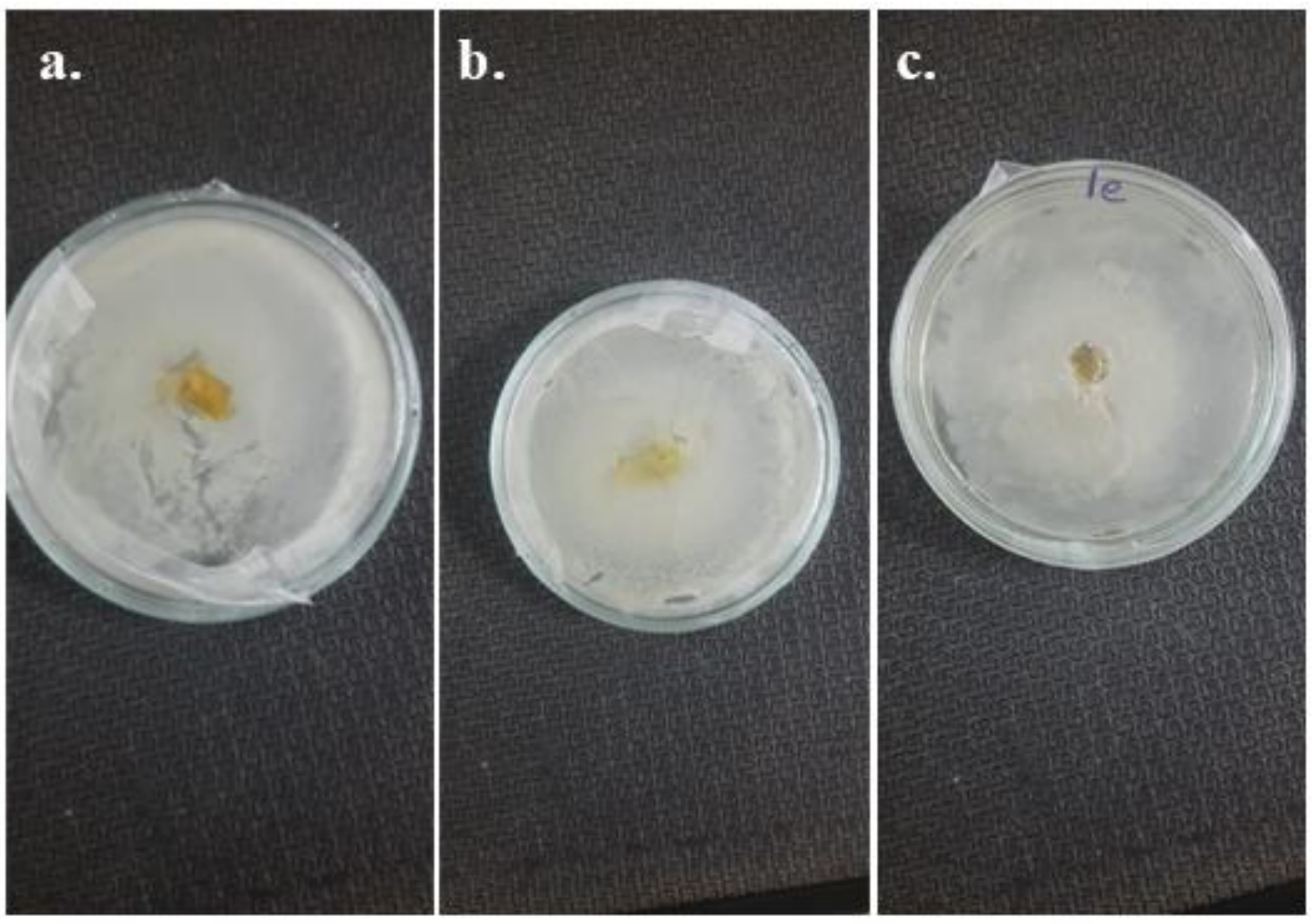

Fig. S4. Microbial Degradation studies of **a**. GC, **b**. GCγ-100 and **c**. GCγ-400 in *E. Coli.*

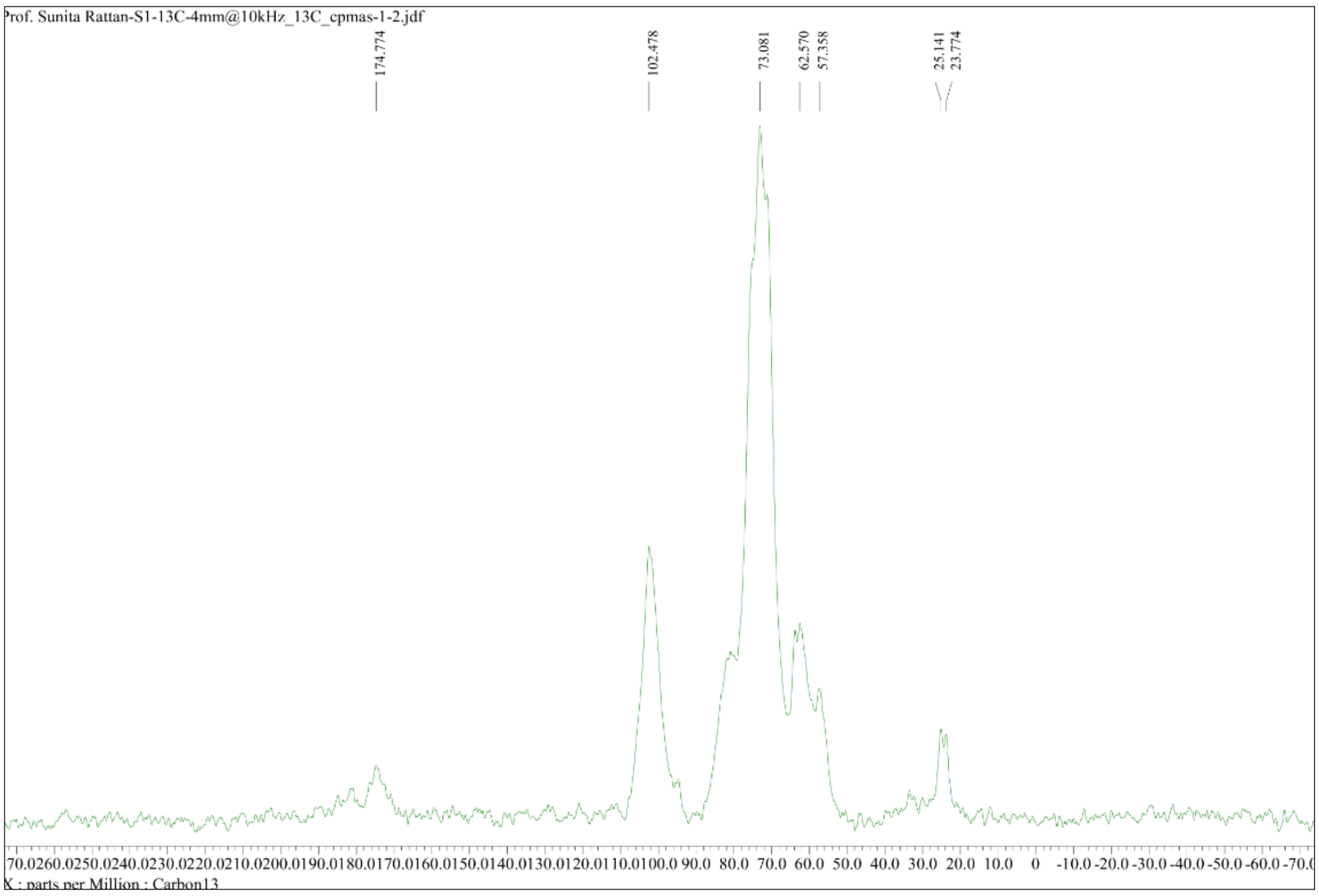

Fig. S5. ^{13}C solid state NMR of GC

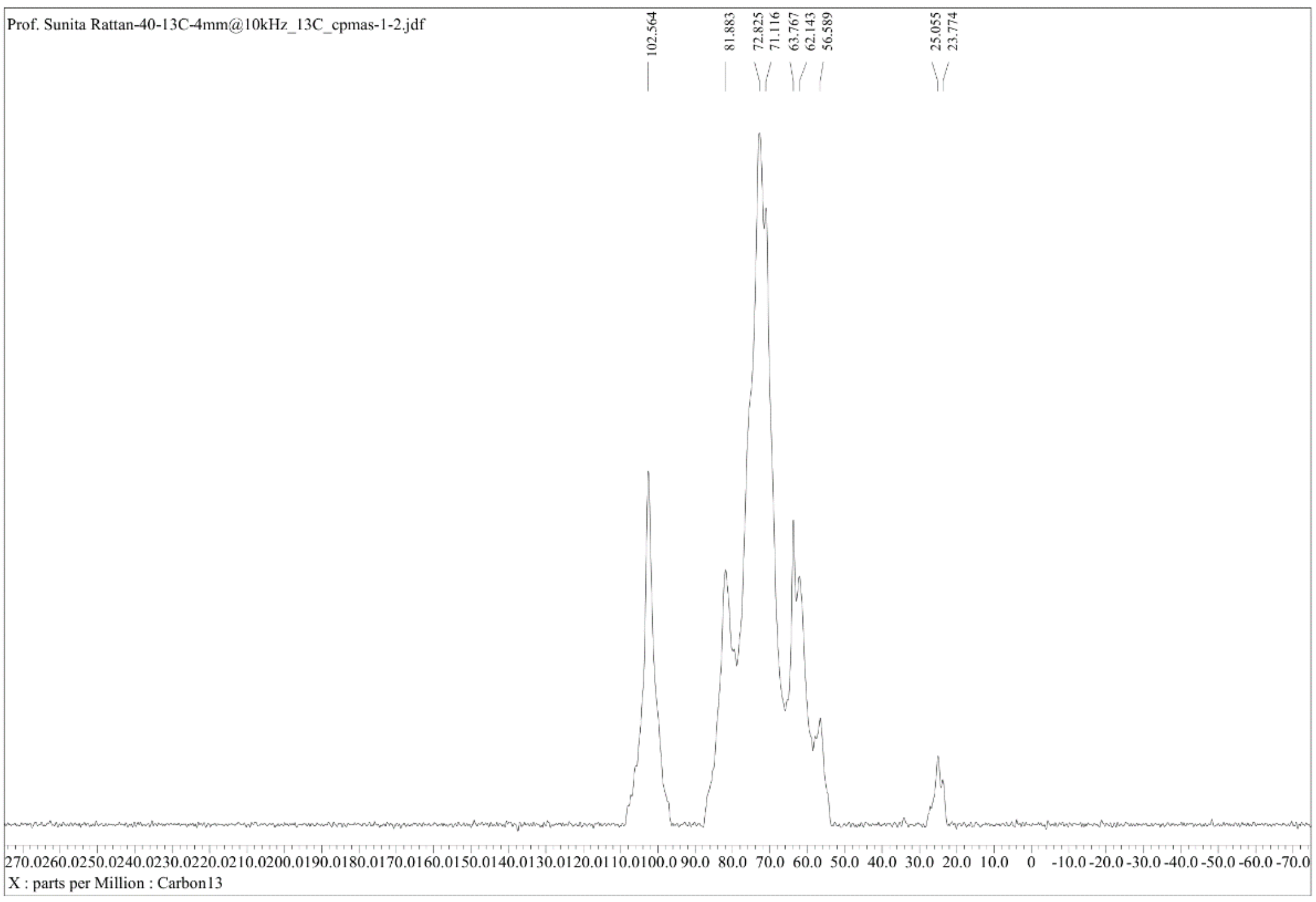

Fig. S6. ^{13}C solid state NMR of GCγ

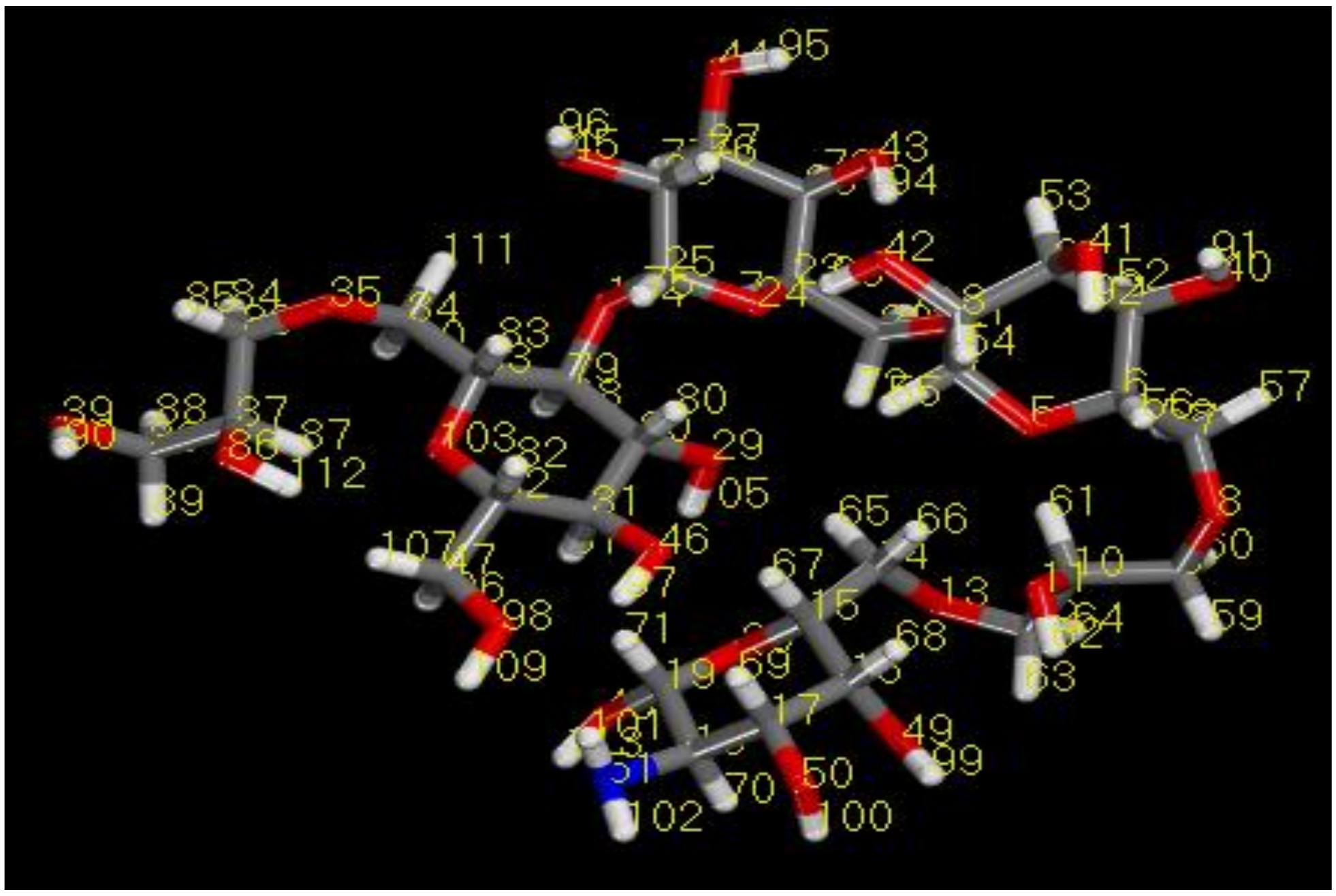

Fig. S7. Labelled structure of GC

Table S3. Hirshfeld Charge and Mayer Bond order for GC.

	Charge		Bond Order
C1	0.0386	C1-C2	1.1426
C2	0.0472	C1-C3	-0.3007
C3	0.0382	C2-C3	1.3692
C4	0.1023	C1-C4	-0.1046
O5	-0.116	C2-C4	-0.1788
C6	0.0419	C3-C4	1.4184
C7	0.0152	C1-O5	-0.1213
O8	-0.1514	C2-O5	-0.1271
C9	0.0115	C3-O5	-0.2429
C10	0.0366	C4-O5	0.8034
O11	-0.1951	C1-C6	1.3237
C12	0.0059	C2-C6	-0.2503

O13	-0.1482	C3-C6	-0.2064
C14	0.0123	C4-C6	-0.193
C15	0.041	O5-C6	0.8299
C16	0.0384	C1-C7	-0.4012
C17	0.0354	C2-C7	0.1269
C18	0.0067	C3-C7	0.1185
C19	0.0988	O5-C7	-0.2811
O20	-0.1433	C6-C7	1.0126
O21	-0.1379	C6-O8	-0.2997
C22	0.0182	C7-O8	0.8321
C23	0.0447	C7-C9	-0.1728
O24	-0.0865	O8-C9	0.7154
C25	0.1049	O5-C10	-0.201
C26	0.036	C7-C10	-0.134
C27	0.0378	O8-C10	-0.1334
C28	0.0398	C9-C10	1.0082
O29	-0.1728	C10-O11	0.6441
C30	0.0394	C9-C12	-0.3337
C31	0.0402	C10-C12	1.1453
C32	0.0446	C10-O13	-0.137
C33	0.0438	C12-O13	0.7266
C34	0.0114	C9-C14	0.1844
O35	-0.1505	C10-C14	-0.1637
C36	0.0112	O13-C14	0.7877
C37	0.0372	C12-C15	-0.1237
C38	0.0123	O13-C15	-0.1598
O39	-0.2195	C14-C15	1.3585
O40	-0.2167	C14-C16	-0.3468
O41	-0.1819	C15-C16	1.5922
O42	-0.2025	C15-C17	-0.4523
O43	-0.2194	C16-C17	1.3275
O44	-0.2201	C14-C18	0.1714
O45	-0.1933	C15-C18	-0.3561
O46	-0.1863	C16-C18	-0.2175

C47	0.0232	C17-C18	1.3328
O48	-0.2207	C15-C19	-0.3391
O49	-0.1884	C16-C19	-0.3621
O50	-0.2183	C17-C19	-0.2496
N51	-0.2022	C18-C19	1.3525
H52	0.0299	C14-O20	-0.4161
H53	0.0448	C15-O20	0.9641
H54	0.0284	C17-O20	-0.1049
H55	0.0337	C18-O20	-0.1521
H56	0.0237	C19-O20	0.8492
H57	0.0365	C1-O21	-0.1876
H58	0.0322	C2-O21	-0.1466
H59	0.0355	C3-O21	-0.2076
H60	0.0289	C4-O21	0.9617
H61	0.0205	C6-O21	-0.1909
H62	0.1321	C4-C22	-0.3092
H63	0.0108	O5-C22	-0.1079
H64	0.0361	C6-C22	0.1147
H65	0.0267	O21-C22	0.6961
H66	0.0265	C3-C23	0.1425
H67	0.0143	C4-C23	-0.1625
H68	0.0272	C22-C23	0.9255
H69	0.0297	C3-O24	-0.1983
H70	0.0317	C4-O24	-0.1152
H71	-0.0006	C22-O24	-0.3462
H72	0.0304	C23-O24	0.9211
H73	0.0477	O24-C25	0.7698
H74	0.0334	C22-C26	0.1934
H75	0.0308	C23-C26	-0.1899
H76	0.0355	O24-C26	-0.221
H77	0.0374	C25-C26	1.1945
H78	0.0384	C22-C27	0.127
C79	0.0435	C23-C27	-0.3271
H80	0.0251	O24-C27	-0.1762

H81	0.0207	C25-C27	-0.1401
H82	0.0327	C26-C27	1.3448
H83	0.0267	C22-C28	-0.4027
H84	0.0293	C23-C28	1.2798
H85	0.0408	C25-C28	-0.3668
O86	-0.2234	C26-C28	-0.2072
H87	0.019	C27-C28	1.2636
H88	0.0396	C15-O29	-0.1908
H89	0.0245	C22-O29	-0.1029
H90	0.1383	O24-O29	0.1568
H91	0.1114	C19-C30	0.1729
H92	0.1456	C25-C30	-0.2695
H93	0.0861	C26-C30	0.1208
H94	0.0891	O29-C30	0.544
H95	0.1254	C18-C31	0.1191
H96	0.1524	C19-C31	-0.2518
H97	0.1064	O20-C31	0.1149
O98	-0.1663	O29-C31	-0.331
H99	0.1314	C30-C31	1.4173
H100	0.1325	C19-C32	0.1272
H101	0.0722	O29-C32	0.1577
H102	0.0961	C30-C32	-0.4616
O103	-0.1134	C31-C32	1.3735
O104	-0.1151	C25-C33	-0.2674
H105	0.1344	O29-C33	0.1382
H106	0.0361	C30-C33	-0.5022
H107	0.0407	C31-C33	-0.4146
H108	0.0349	C32-C33	-0.3458
H109	0.1569	C30-C34	0.1067
H110	0.0307	C31-C34	0.1597
H111	0.0298	C33-C34	0.908
H112	0.1101	C33-O35	-0.3422
H113	0.1259	C34-O35	0.7535
		C34-C36	-0.2188

O35-C36	0.7116
C34-C37	-0.1895
O35-C37	-0.1056
C36-C37	1.0362
C36-C38	-0.1929
C37-C38	0.9919
C38-O39	0.7571
C1-O40	0.6998
C2-O40	-0.1902
C4-O40	0.1204
O5-O40	0.14
C6-O40	-0.2463
C7-O40	-0.1141
C2-O41	0.7608
C3-O41	-0.2893
C4-O41	0.1085
O5-O41	0.1155
C2-O42	-0.3596
C3-O42	0.8776
C4-O42	-0.1856
C6-O42	0.1219
C23-O43	-0.123
C28-O43	0.8703
C23-O44	0.1126
O24-O44	0.132
C25-O44	0.1013
C26-O44	-0.231
C27-O44	0.6528
C28-O44	-0.2423
C23-O45	0.1024
O24-O45	0.1014
C25-O45	-0.1887
C26-O45	0.7639
C27-O45	-0.232

C15-O46	-0.1928
C17-O46	-0.1067
C30-O46	-0.2327
C31-O46	0.6709
C32-O46	-0.2914
C33-O46	0.18
C30-C47	0.1477
C31-C47	-0.3797
C32-C47	0.9913
C16-O48	0.1262
C18-O48	-0.1924
C19-O48	0.8834
O20-O48	-0.169
C14-O49	-0.1101
C15-O49	-0.1598
C16-O49	0.8482
C18-O49	-0.1186
C15-O50	0.1167
C16-O50	-0.191
C17-O50	0.7674
C18-O50	-0.1996
O20-O50	0.1046
C15-N51	0.1374
C16-N51	0.1006
C17-N51	-0.1958
C18-N51	0.8355
C19-N51	-0.2045
C1-H52	1.3059
C2-H52	-0.1482
C3-H52	-0.146
O21-H52	0.1689
C1-H53	-0.1559
C2-H53	1.1857
C1-H54	-0.1097

C3-H54	1.4413
C1-H55	0.116
C3-H55	-0.2633
C4-H55	1.018
C6-H55	-0.103
C23-H55	-0.1284
C4-H56	-0.1033
C6-H56	1.3416
C6-H57	-0.1372
C7-H57	1.0739
C1-H58	-0.1365
C7-H58	1.1731
C10-H58	-0.1109
C9-H59	1.1182
C9-H60	1.0296
C4-H61	-0.1636
O5-H61	0.4631
C6-H61	-0.2558
C9-H61	-0.1726
C10-H61	1.1207
C12-H61	-0.1893
C14-H61	-0.2664
O11-H62	1.0004
C12-H63	1.0641
O49-H63	0.2292
C12-H64	1.1341
O13-H65	-0.1176
C14-H65	1.1821
C15-H65	-0.3764
C22-H65	-0.1035
C4-H66	-0.1318
C14-H66	1.0413
C15-H66	-0.2323
C16-H66	-0.1569

C19-H66	0.1075
H55-H66	0.2272
H61-H66	0.2052
C14-H67	-0.3672
C15-H67	1.1184
C16-H67	-0.3555
C17-H67	-0.1318
C18-H67	-0.1613
C19-H67	-0.1383
O29-H67	0.3081
C30-H67	-0.2533
C31-H67	-0.1608
O46-H67	0.3842
H66-H67	-0.1416
O11-H68	0.1996
C14-H68	-0.1236
C15-H68	-0.1269
C16-H68	1.1106
C17-H68	-0.2299
C19-H68	0.1834
H65-H68	0.1179
H67-H68	-0.1567
C17-H69	1.3397
H67-H69	0.1503
C18-H70	1.2886
H67-H70	0.12
C15-H71	-0.219
C17-H71	-0.1199
C18-H71	-0.2904
C19-H71	1.1684
O20-H71	-0.1856
C30-H71	-0.1572
C31-H71	-0.3483
C32-H71	-0.1672

O48-H71	-0.1758
N51-H71	-0.1038
H67-H71	0.1641
C14-H72	-0.103
C22-H72	1.0249
C23-H72	-0.2128
O29-H72	0.1051
H55-H72	0.1623
H65-H72	0.2015
C22-H73	1.097
C23-H73	-0.1171
C22-H74	-0.1829
C23-H74	1.3774
C26-H74	-0.1348
C27-H74	-0.1082
C28-H74	-0.1468
C23-H75	-0.1472
C25-H75	1.2406
C26-H75	-0.2653
C28-H75	0.1906
C30-H75	-0.2247
C33-H75	-0.1334
C26-H76	1.3747
C23-H77	-0.107
C27-H77	1.2564
C28-H77	-0.124
C23-H78	-0.1516
C25-H78	0.1216
C27-H78	-0.1368
C28-H78	1.1276
O24-C79	-0.2697
C25-C79	-0.1475
C28-C79	0.1207
O29-C79	-0.2507

C30-C79	1.6211
C31-C79	-0.3609
C32-C79	-0.3322
C33-C79	1.3914
C34-C79	-0.4949
O35-C79	0.1304
C47-C79	0.1312
H74-C79	-0.1093
C30-H80	1.2788
C31-H80	-0.1851
C32-H80	-0.1231
C47-H80	0.1211
C19-H81	-0.256
C30-H81	-0.1291
C31-H81	1.4584
C47-H81	-0.1463
H67-H81	-0.118
H71-H81	0.5235
C79-H81	-0.1953
C30-H82	-0.1013
C32-H82	1.4132
C33-H82	-0.1199
C30-H83	-0.1549
C31-H83	-0.1106
C32-H83	-0.1245
C33-H83	1.524
H75-H83	0.1401
C36-H84	1.0679
C36-H85	1.0378
C37-O86	0.9245
C33-H87	-0.1487
C37-H87	1.1511
C38-H87	-0.1271
C38-H88	1.0718

C38-H89	1.0819
O39-H90	0.9811
O40-H91	0.9745
O41-H91	0.1231
O41-H92	0.9312
C1-H93	0.1237
C2-H93	-0.1779
C3-H93	-0.3158
C4-H93	0.1215
C23-H93	-0.1662
O24-H93	0.6135
C25-H93	-0.2335
C26-H93	-0.1309
O41-H93	0.11
O42-H93	0.6521
H54-H93	-0.1164
H55-H93	0.116
C3-H94	-0.108
O21-H94	0.1562
C27-H94	-0.139
C28-H94	-0.1598
O42-H94	0.3522
O43-H94	0.6184
H53-H94	0.1281
H74-H94	0.1276
H77-H94	0.1163
H93-H94	0.125
C26-H95	-0.1156
O43-H95	0.164
O44-H95	0.9874
O45-H96	1.0284
C30-H97	-0.1278
C31-H97	-0.1017
O46-H97	0.701

H67-H97	-0.2331
C31-O98	-0.1175
C32-O98	-0.2234
C47-O98	0.6539
H97-O98	0.3364
C16-H99	-0.1451
C17-H99	-0.1142
O49-H99	0.8842
C16-H100	-0.1199
O50-H100	0.9751
N51-H101	0.9318
C18-H102	-0.1365
N51-H102	1.158
O29-O103	0.1228
C30-O103	-0.1369
C31-O103	-0.1115
C32-O103	0.8579
C33-O103	0.8458
C34-O103	-0.2894
O46-O103	0.1654
C47-O103	-0.4619
C79-O103	-0.2347
H87-O103	0.224
O98-O103	0.1429
C22-O104	0.1014
C23-O104	-0.2746
C25-O104	1.1318
C26-O104	-0.2089
C27-O104	-0.2177
C30-O104	-0.2704
C32-O104	0.1589
C33-O104	-0.1836
C34-O104	-0.215
H74-O104	0.2203

C79-O104	0.645
O103-O104	0.1109
C19-H105	-0.1349
O29-H105	0.7265
C31-H105	-0.1098
C32-H105	0.1532
C33-H105	0.1278
H65-H105	0.1013
H67-H105	0.2657
C79-H105	-0.2291
C47-H106	1.1446
C32-H107	-0.1821
C47-H107	1.0243
C25-H108	-0.1209
C31-H108	-0.1051
H75-H108	0.1033
C79-H108	1.3028
O98-H109	0.9638
H101-H109	0.1023
C34-H110	1.3668
C37-H110	-0.1084
C79-H110	-0.1293
O103-H110	-0.126
C32-H111	0.1243
C33-H111	-0.2138
C34-H111	0.955
O35-H111	-0.1004
O45-H111	0.1114
O104-H111	0.2136
C37-H112	-0.131
O86-H112	0.7661
O103-H112	0.2188
H107-H112	0.1713
O48-H113	0.9302

		N51-H113	0.2926

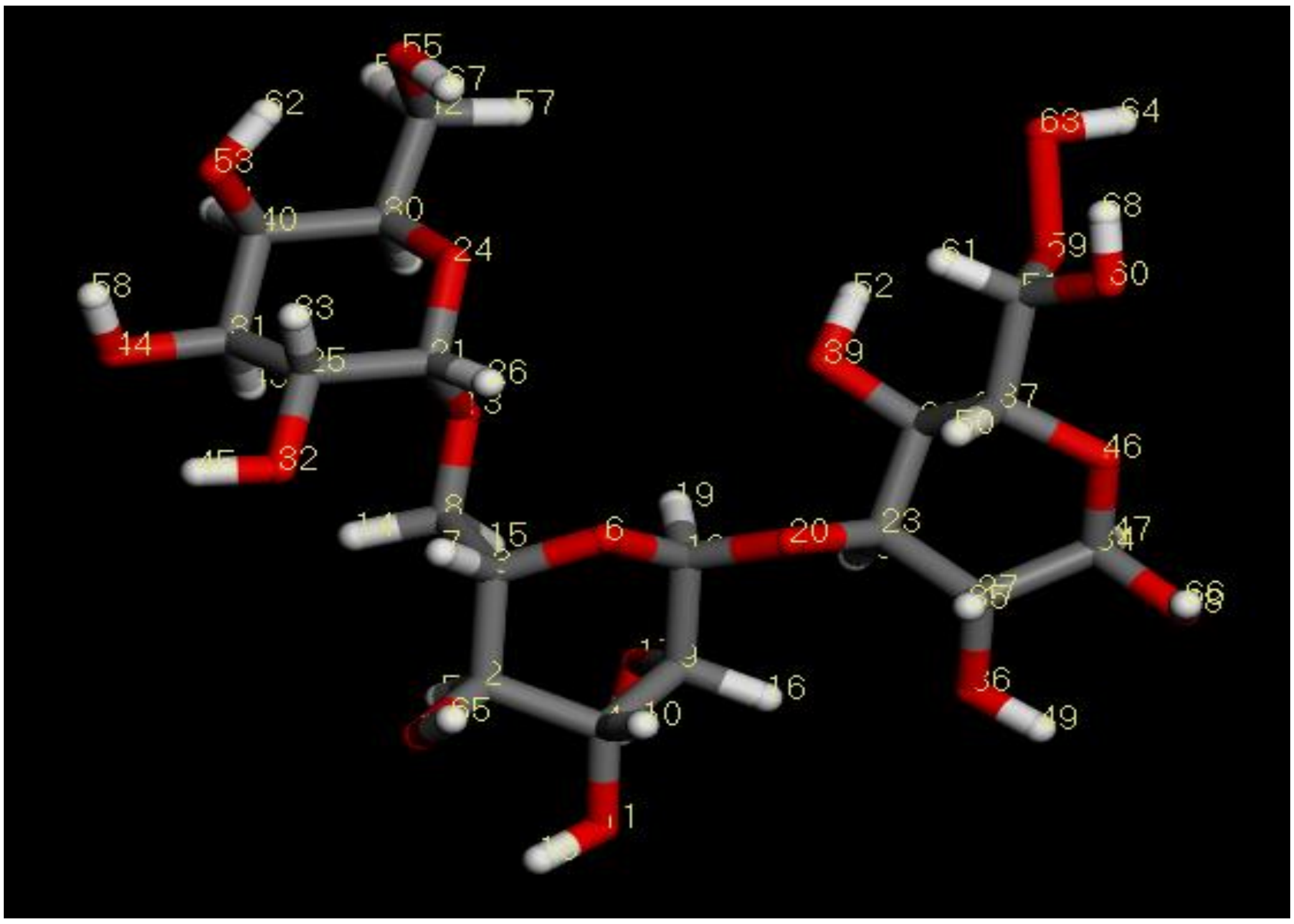

Fig. S8. Labelled structure of γ-G

Table S4. Hirshfeld Charge and Mayer Bond order for γ-G.

	Charge		Bond Order
O1	-0.2061	O1-C2	0.7489
C2	0.0420	O1-C3	-0.2275
C3	0.0412	C2-C3	1.331
C4	0.0346	O1-C4	-0.1692
H5	0.0343	C2-C4	1.1658
O6	-0.1365	C3-C4	-0.3695
H7	0.0275	C2-H5	1.3533
C8	0.0059	C2-O6	-0.1237
C9	0.0374	C3-O6	0.88

H10	0.0278	C2-H7	-0.1193
O11	-0.2076	C3-H7	1.2149
C12	0.111	O6-H7	-0.1107
O13	-0.1317	C2-C8	-0.5365
H14	0.0265	C3-C8	1.1764
H15	0.0295	C2-C9	-0.147
H16	0.0256	C3-C9	-0.2233
O17	-0.1998	C4-C9	1.1279
H18	0.1412	H5-C9	-0.1128
H19	0.0254	O6-C9	-0.2179
O20	-0.1324	H7-C9	0.101
C21	0.111	C3-H10	-0.1505
H22	0.1422	C4-H10	1.1901
C23	0.0504	C2-O11	-0.2081
O24	-0.1391	C3-O11	0.1151
C25	0.0296	C4-O11	0.8016
H26	0.038	C9-O11	-0.1495
C27	0.0362	O1-C12	0.1095
C28	0.0401	C2-C12	-0.2779
H29	0.0406	C3-C12	-0.1851
C30	0.0432	C4-C12	-0.2254
C31	0.039	O6-C12	0.962
O32	-0.1866	H7-C12	-0.1079
H33	0.0223	C8-C12	-0.169
C34	0.1048	C9-C12	1.5221
H35	0.0261	C3-O13	-0.2092
O36	-0.1949	C4-O13	0.1227
C37	0.0451	C8-O13	0.5748
H38	0.031	C12-O13	-0.1383
O39	-0.1952	C3-H14	-0.1627
C40	0.0417	C8-H14	1.0811
H41	0.0359	C8-H15	0.977
C42	0.0249	O13-H15	-0.1385
H43	0.0358	C3-H16	0.1746

O44	-0.2038
H45	0.1379
O46	-0.1381
H47	0.0352
O48	-0.1982
H49	0.141
H50	0.0315
C51	0.0884
H52	0.1415
O53	-0.2179
H54	0.0313
O55	-0.1728
H56	0.0506
H57	0.0454
H58	0.1377
O59	-0.1297
O60	-0.2171
H61	0.0272
H62	0.1052
O63	-0.2194
H64	0.1265
H65	0.1475
H66	0.1493
H67	0.1529
H68	0.1404

C4-H16	-0.2551
C9-H16	1.0371
O6-O17	0.1218
C9-O17	0.8411
C12-O17	-0.3739
H15-O17	0.1935
O1-H18	0.1218
O11-H18	1.0021
C3-H19	-0.1694
O6-H19	-0.1004
C12-H19	1.4198
C2-O20	0.123
O6-O20	-0.3087
C9-O20	-0.2891
C12-O20	0.8836
O17-O20	0.1482
H19-O20	-0.1126
C2-C21	0.1099
C3-C21	-0.1992
O6-C21	-0.1821
C8-C21	-0.2366
O13-C21	1.1331
H14-C21	-0.1087
O11-H22	0.1356
O17-H22	0.965
C3-C23	0.1202
C4-C23	0.1367
C9-C23	-0.4417
C12-C23	-0.4345
O17-C23	0.1015
H19-C23	-0.1568
O20-C23	0.9401
O13-O24	-0.1682
C21-O24	0.6549

H7-C25	-0.1509
C8-C25	-0.123
C21-C25	1.3742
O24-C25	-0.2468
C21-H26	1.1621
O24-H26	-0.1096
C25-H26	-0.1242
H16-C27	-0.1595
O20-C27	-0.4302
C23-C27	1.2851
C9-C28	0.1248
C12-C28	-0.2019
H19-C28	-0.1007
C23-C28	1.1096
C27-C28	-0.3666
H16-H29	0.1637
C23-H29	1.4118
C28-H29	-0.1305
O13-C30	-0.231
O24-C30	0.9447
C25-C30	-0.2101
O13-C31	-0.1119
C25-C31	1.2899
C30-C31	-0.2907
C3-O32	-0.1467
H7-O32	0.1977
H14-O32	0.1209
C21-O32	-0.2612
O24-O32	0.1445
C25-O32	0.7354
C30-O32	0.1301
C31-O32	-0.2379
C8-H33	0.1071
C25-H33	1.2721

C30-H33	-0.1078
O20-C34	0.1359
C23-C34	-0.3466
C27-C34	1.2637
C28-C34	-0.13
H29-C34	-0.1026
C27-H35	1.2955
C28-H35	-0.1377
C23-O36	-0.3252
C27-O36	0.7662
C28-O36	0.1486
C34-O36	-0.2233
O20-C37	-0.2053
C23-C37	-0.2226
C27-C37	-0.1517
C28-C37	1.3925
C34-C37	-0.3338
O36-C37	0.1225
C27-H38	-0.146
C28-H38	1.2514
H35-H38	0.1099
C23-O39	-0.2516
C27-O39	0.1287
C28-O39	0.839
C34-O39	0.124
C37-O39	-0.1716
C8-C-40	0.1308
C21-C40	-0.351
C25-C40	-0.1662
H26-C40	0.1333
C30-C40	1.197
C31-C40	1.0717
O13-H41	0.1038
C30-H41	1.2852

O24-C42	-0.2854
C25-C42	0.1256
C30-C42	0.8747
C31-C42	0.1433
C40-C42	-0.3091
C8-H43	-0.1131
H14-H43	0.1175
C31-H43	1.3024
C40-H43	-0.1294
O24-O44	0.1099
C25-O44	-0.1676
C30-O44	0.1092
C31-O44	0.6869
C40-O44	-0.1321
O32-H45	1.0035
O44-H45	0.1463
O20-O46	0.1238
C23-O46	-0.1418
C27-O46	-0.1487
C28-O46	-0.161
C34-O46	0.8575
O36-O46	0.1099
C37-O46	0.8848
O39-O46	0.1244
C34-H47	1.2152
C27-O48	-0.1759
C34-O48	0.7851
O46-O48	-0.1128
O36-H49	0.9963
O48-H49	0.1332
C34-H50	-0.1004
C37-H50	1.2736
C28-C51	-0.3196
C37-C51	0.9234

O46-C51	-0.1732
O39-H52	0.849
C25-O53	-0.1452
C40-O53	0.8728
C21-H54	0.1289
C30-H54	-0.1301
C31-H54	-0.1355
C40-H54	1.1467
C42-O55	0.7274
C42-H56	1.085
C30-H57	-0.1548
C42-H57	1.083
O44-H58	0.9842
O53-H58	0.1198
C28-O59	-0.1279
C37-O59	-0.101
C51-O59	0.9614
H52-O59	0.1399
C37-O60	-0.196
C51-O60	0.8563
C37-H61	-0.1102
C51-H61	1.1463
O53-H62	0.8581
O55-H62	0.1751
O59-O63	0.5886
O63-H64	1.0296
O1-H65	0.9922
O48-H66	0.9789
O24-H67	0.1041
O55-H67	0.9309
O60-H68	0.9762

References:

1 R. Katharia, R. Chaudhary and P. Agarwal, Prestorage gamma irradiation induces oxidative injury to red cells, , *Transfus. Apher. Sci.*, 2013, **48**, 39–43.

2 M. Shahid, S. A. Bukhari, Y. Gul, H. Munir, F. Anjum, M. Zuber, T. Jamil and K. M. Zia, Graft polymerization of guar gum with acryl amide irradiated by microwaves for colonic drug delivery, , *Int. J. Biol. Macromol.*, 2013, **62**, 172–179.

3 S. Liyanage, S. S. Rumi and N. Abidi, Preparation and optimization of guar-based rolling films with enhanced physicochemical properties, , *Ind. Crops Prod.*, 2026, **240**, 122644.

4 G. Chen, J. Mi, X. Wu, C. Luo, J. Li, Y. Tang and J. Li, Structural features and bioactivities of the chitosan, , *Int. J. Biol. Macromol.*, 2011, **49**, 543–547.

5 A. P. Safronov, L. V. Adamova and G. V. Kurlyandskaya, Flory–Huggins Parameters of Guar Gum, Xanthan Gum, Agarose, and Gellan Gum in Aqueous Solutions, , *Polym. Sci. Ser. A*, 2019, **61**, 29–38.

6 M. R. Kasaai, Calculation of Mark–Houwink–Sakurada (MHS) equation viscometric constants for chitosan in any solvent–temperature system using experimental reported viscometric constants data, , *Carbohydr. Polym.*, 2007, **68**, 477–488.

7 C. N. Costa, V. G. Teixeira, M. C. Delpech, J. V. S. Souza and M. A. S. Costa, Viscometric study of chitosan solutions in acetic acid/sodium acetate and acetic acid/sodium chloride, , *Carbohydr. Polym.*, 2015, **133**, 245–250.

8 P. J. do Amaral Sobral, G. Gebremariam, F. Drudi, A. C. De Aguiar Saldanha Pinheiro, S. Romani, P. Rocculi and M. Dalla Rosa, Rheological and Viscoelastic Properties of Chitosan Solutions Prepared with Different Chitosan or Acetic Acid Concentrations, , *Foods*, 2022, **11**, 2692.

9 K. Jumel, S. E. Harding and J. R. Mitchell, Effect of gamma irradiation on the macromolecular integrity of guar gum, , *Carbohydr. Res.*, 1996, **282**, 223–236.

10 J. Choi, H.-S. Lee, J.-H. Kim, K.-W. Lee, Y.-J. Chung, M.-W. Byun and J.-W. Lee, Effect of electron beam irradiation on the viscosity of carboxymethylcellulose solution, , *Nucl. Instruments Methods Phys. Res. Sect. B Beam Interact. with Mater. Atoms*, 2008, **266**, 5068–5071.
